# Identification and Roles of Photosystem II Assembly, Stability, and Repair Factors in Arabidopsis

**DOI:** 10.3389/fpls.2016.00168

**Published:** 2016-02-16

**Authors:** Yan Lu

**Affiliations:** Department of Biological Sciences, Western Michigan UniversityKalamazoo, MI, USA

**Keywords:** Photosystem II assembly, Photosystem II stability, Photosystem II repair, *Arabidopsis thaliana*, identification and roles

## Abstract

Photosystem II (PSII) is a multi-component pigment-protein complex that is responsible for water splitting, oxygen evolution, and plastoquinone reduction. Components of PSII can be classified into core proteins, low-molecular-mass proteins, extrinsic oxygen-evolving complex (OEC) proteins, and light-harvesting complex II proteins. In addition to these PSII subunits, more than 60 auxiliary proteins, enzymes, or components of thylakoid protein trafficking/targeting systems have been discovered to be directly or indirectly involved in *de novo* assembly and/or the repair and reassembly cycle of PSII. For example, components of thylakoid-protein-targeting complexes and the chloroplast-vesicle-transport system were found to deliver PSII subunits to thylakoid membranes. Various auxiliary proteins, such as PsbP-like (Psb stands for PSII) and light-harvesting complex-like proteins, atypical short-chain dehydrogenase/reductase family proteins, and tetratricopeptide repeat proteins, were discovered to assist the *de novo* assembly and stability of PSII and the repair and reassembly cycle of PSII. Furthermore, a series of enzymes were discovered to catalyze important enzymatic steps, such as C-terminal processing of the D1 protein, thiol/disulfide-modulation, peptidylprolyl isomerization, phosphorylation and dephosphorylation of PSII core and antenna proteins, and degradation of photodamaged PSII proteins. This review focuses on the current knowledge of the identities and molecular functions of different types of proteins that influence the assembly, stability, and repair of PSII in the higher plant *Arabidopsis thaliana*.

## Introduction

Photosystem II (PSII) is a multi-subunit pigment-protein complex found in thylakoid membranes of oxygenic photosynthetic organisms, including cyanobacteria, algae, and plants (Nickelsen and Rengstl, 2013; Järvi et al., 2015). Driven by light, PSII catalyzes electron transfer from water to plastoquinone. Therefore, PSII is also known as a water-plastoquinone oxidoreductase. Proteomics, X-ray crystallography, and single-particle electron cryo-microscopy studies revealed that PSII components include core proteins, low-molecular-mass (LMM, i.e., < 10 kDa) proteins, extrinsic oxygen-evolving complex (OEC) proteins, and light-harvesting complex (LHC) proteins (da Fonseca et al., 2002; Kashino et al., 2002; Liu et al., 2004; Aro et al., 2005; Nield and Barber, 2006; Umena et al., 2011; Suga et al., 2015). Except for some minor differences in the composition of LMM proteins, the core of PSII is conserved from cyanobacteria to land plants (Umena et al., 2011; Nickelsen and Rengstl, 2013). Proteins that form the PSII core complex in land plants include PSII reaction center core proteins D1 and D2 (i.e., PsbA and PsbD; Psb stands for PSII), core antenna proteins CP43 and CP47 (i.e., PSII chlorophyll proteins of 43 and 47 kDa, also known as PsbC and PsbB, respectively), cytochrome *b*_559_ subunits alpha and beta (i.e., PsbE and PsbF), and LMM proteins PsbH, PsbI, PsbJ, PsbK, PsbL, PsbM, PsbR, PsbTc (chloroplast-encoded PSII protein T), PsbTn (nuclear-encoded PSII protein T), PsbW, PsbX, PsbY, and PsbZ (Nickelsen and Rengstl, 2013). Due to the loss of PsbU and PsbV during green plant evolution, cyanobacterial OEC has PsbO, PsbP, PsbQ, PsbU, and PsbV subunits but land plant OEC only contains PsbO, PsbP, and PsbQ subunits (Thornton et al., 2004; Bricker et al., 2012). The PSII-light-harvesting antenna in cyanobacteria is made of phycobilisomes, which are attached to the cytoplasmic side of PSII (Liu et al., 2005). The PSII-light-harvesting antenna (i.e., light-harvesting complex II, abbreviated as LHCII) in land plants is an integral membrane complex. LHCII contains three major trimeric PSII light-harvesting chlorophyll *a/b*-binding (LHCB) proteins LHCB1, LHCB2, and LHCB3 and three minor monomeric LHCB proteins LHCB4, LHCB5, and LHCB6 (Jansson, 1999; Liu et al., 2004). In addition to PSII subunits, more than 60 auxiliary proteins or enzymes have been found to be involved in the assembly, stability, and repair of PSII complexes (Nixon et al., 2010; Nickelsen and Rengstl, 2013; Järvi et al., 2015). This article focuses on the identification and roles of different types of proteins that influence the assembly, stability, and repair of PSII in the higher plant *Arabidopsis thaliana*.

## *De novo* assembly of PSII

**Figure 1:**
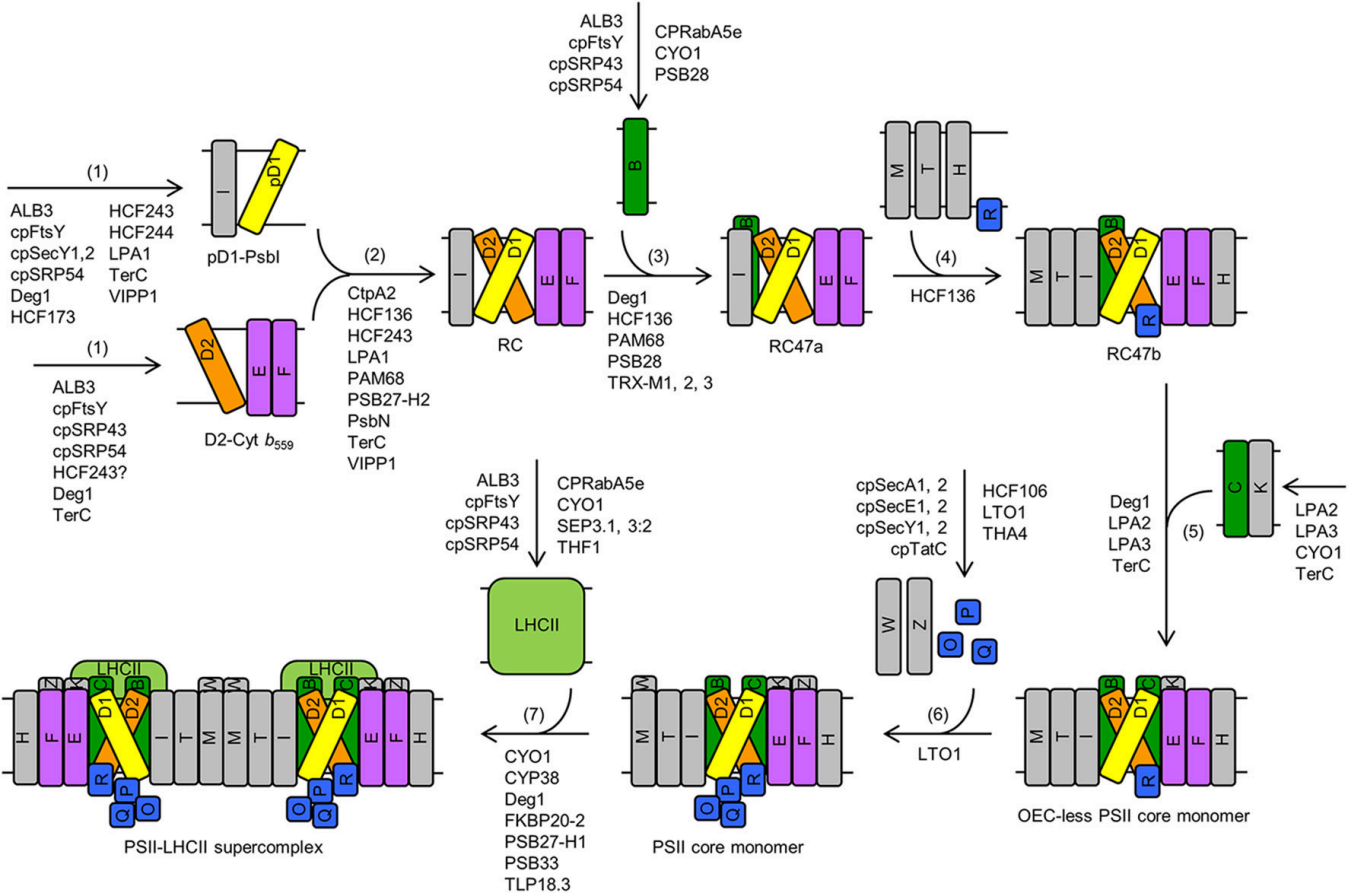
***De novo* assembly of PSII in Arabidopsis**. The major steps include: (1) assembly of precursor D1-PsbI (pD1-PsbI) and D2-cytochrome *b*_559_ (D2-Cyt *b*_559_) precomplexes, (2) assembly of the minimal reaction-center complex (RC), which lacks CP47 and CP43, (3) assembly of the reaction-center complex (RC47a) that contains CP47 but lacks CP43, (4) incorporation of LMM subunits, such as PsbH, PsbM, PsbT, and PsbR, to form RC47b, (5) incorporation of CP43, along with LMM subunit PsbK, to form the OEC-less PSII core monomer, (6) assembly of the oxygen-evolving complex (OEC) and additional LMM subunits, such as PsbW and PsbZ, to form the PSII core monomer, and (7) dimerization and formation of the PSII-light-harvesting complex II (LHCII) supercomplex. Proteins that are involved in these steps are listed. Although RBD1 promotes PSII assembly and/or PSII stability, it is not depicted in this figure because it is not clear which step(s) of *de novo* PSII assembly this protein is involved in. Letters (B, C, D1, D2, E, F, H, I, K, M, O, P, Q, R, T, W, Z) in rectangles represent PSII proteins PsbB (i.e., CP47), PsbC (i.e., CP43), D1, D2, PsbE, PsbF, PsbH, PsbI, PsbK, PsbM, PsbO, PsbP, PsbQ, PsbR, PsbT, PsbW, and PsbZ, respectively. Abbreviations: D2-Cyt *b*_559_, D2-cytochrome *b*_559_ precomplex; LHCII, light-harvesting complex II; OEC, oxygen-evolving complex; pD1, precursor D1; pD1-PsbI, precursor D1-PsbI precomplex; PSII, Photosystem II; RC, PSII minimal reaction-center complex; RC47a, PSII reaction-center complex with CP47, without PsbM, PsbH, PsbT, or PsbR; RC47b, PSII reaction-center complex with CP47, PsbM, PsbH, PsbT, and PsbR. For simplicity, only one name is shown for proteins with multiple names (e.g., “THF1” for THF1/PSB29).

*De novo* (Latin for “anew” or “from the beginning”) PSII assembly is a sequential and highly coordinated process. The principal steps were revealed by the use of radioactive pulse-chase experiments, two-dimensional blue native/sodium dodecyl sulfate-polyacrylamide gel electrophoresis, and subsequent proteomics and mass spectrometry analysis (Aro et al., 2005; Rokka et al., 2005; Boehm et al., 2012a). *De novo* PSII assembly in higher plants include: (1) assembly of the precursor D1-PsbI (pD1-PsbI) and D2-cytochrome *b*_559_ (D2-Cyt *b*_559_) precomplexes, (2) assembly of the minimal reaction-center complex (RC), which lacks CP47 and CP43, (3) assembly of the reaction-center complex (RC47a) that contains CP47 but lacks CP43, (4) incorporation of LMM subunits, such as PsbH, PsbM, PsbT, and PsbR, to form RC47b, (5) incorporation of CP43, along with LMM subunit PsbK, to form the OEC-less PSII monomer, (6) assembly of the OEC and additional LMM subunits, such as PsbW and PsbZ, to form the PSII core monomer, and (7) dimerization and formation of the PSII-LHCII supercomplex (Figure 1; Rokka et al., 2005; Nixon et al., 2010; Komenda et al., 2012a; Nickelsen and Rengstl, 2013). A similar pathway exists in cyanobacteria, algae, and lower plants, suggesting that the core components of PSII and the assembly process of PSII complexes are conserved (Nixon et al., 2010; Komenda et al., 2012a; Nickelsen and Rengstl, 2013). From cyanobacteria to green algae to land plants, the initial assembly steps of photosynthetic complexes appear to be spatially separated from sites of active photosynthesis (Nickelsen and Rengstl, 2013). For instance, in *Chlamydomonas reinhardtii*, initial steps of *de novo* PSII assembly occur in discrete regions near the pyrenoid, called translation zones (Uniacke and Zerges, 2007).

Assembly of LHCII is recently thought to initiate on the chloroplast envelope in developing chloroplasts and on thylakoid membranes in developed chloroplasts (Tanz et al., 2012; Khan et al., 2013). LHCII assembly on the chloroplast envelope consists of four major steps: (1) partial insertion of LHCP (light-harvesting chlorophyll *a/b*-binding protein) apoproteins into the inner chloroplast envelope, (2) binding of chlorophyll to reach a stable conformation in the membrane, (3) insertion of the rest of the protein domains, and (4) further pigment binding and protein assembly into a fully assembled pigment-protein complex (Hoober et al., 2007; Dall'Osto et al., 2015). The pigment-protein complexes on the inner chloroplast envelope can be transferred to thylakoid membranes via the chloroplast-vesicle-transport system, the primary source of lipids and proteins for developing thylakoids in young chloroplasts (Tanz et al., 2012; Khan et al., 2013; Karim et al., 2014). In developed chloroplasts, LHCP proteins are primarily transported and integrated into thylakoid membranes via the chloroplast signal recognition particle (cpSRP) pathway (Cline and Dabney-Smith, 2008; Albiniak et al., 2012; Dall'Osto et al., 2015).

## Damage, repair, and reassembly of PSII

**Figure 2:**
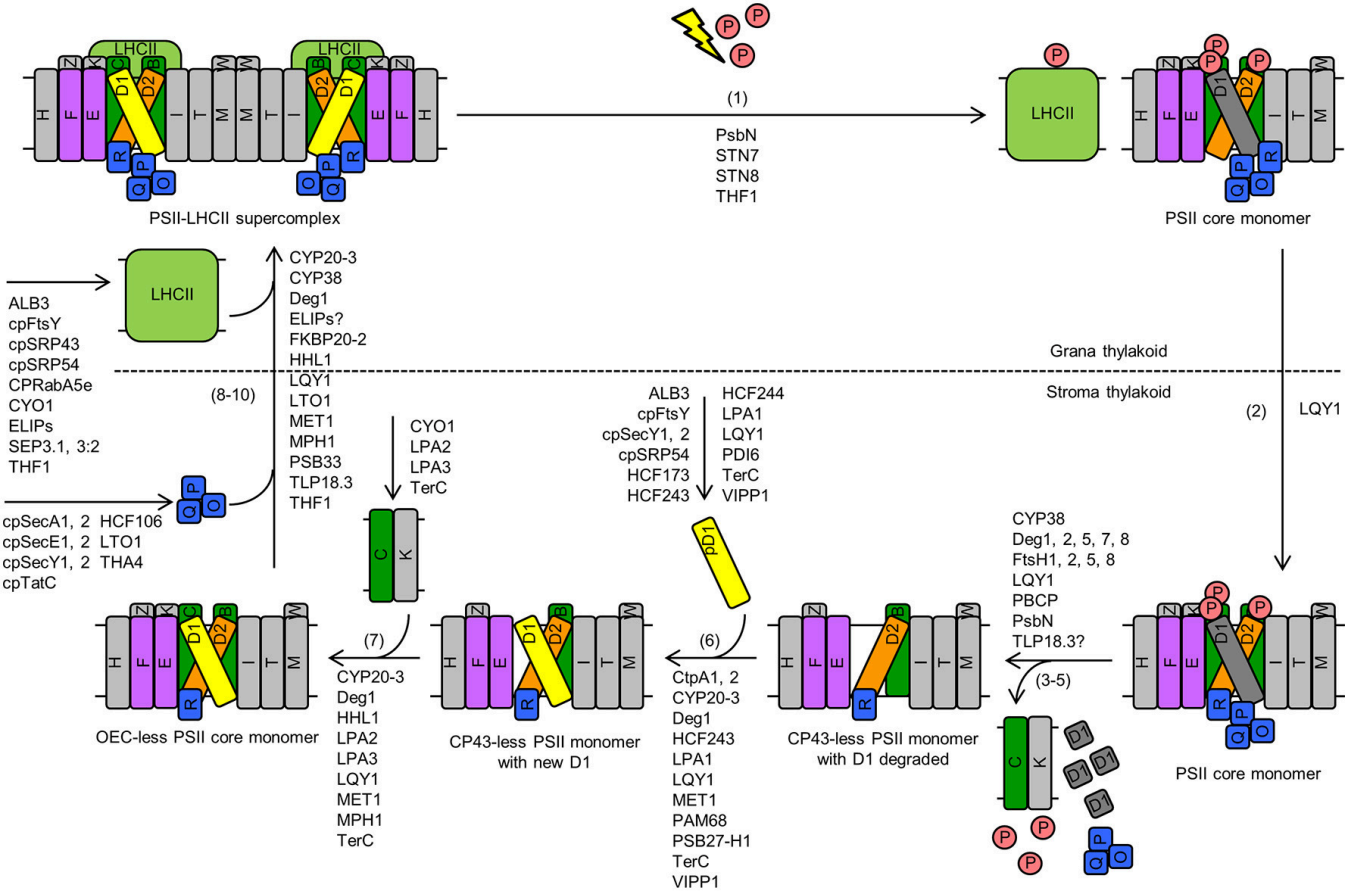
**Damage, repair, and reassembly of PSII in Arabidopsis**. The major steps include: (1) high-light-induced phosphorylation, damage, and disassembly of the PSII-LHCII supercomplex and the PSII core dimer in grana stacks, (2) lateral migration of the PSII core monomer to stroma-exposed thylakoid membranes, (3–5) dephosphorylation, partial disassembly of the PSII core monomer, and degradation of photodamaged D1, (6) synthesis and reassembly of new D1, (7) re-incorporation of CP43, (8) reattachment of OEC, (9) migration of the PSII core monomer back to grana stacks, and (10) dimerization into PSII core dimers and reformation of PSII-LHCII supercomplexes. Proteins that are involved in these steps are listed. Although PPL1 might be involved in PSII repair, it is not depicted in this figure because it is not clear which step(s) of PSII repair this protein is involved in. Letters (B, C, D1, D2, E, F, H, I, K, M, O, P, Q, R, T, W, Z) in rectangles represent PSII proteins PsbB (i.e., CP47), PsbC (i.e., CP43), D1, D2, PsbE, PsbF, PsbH, PsbI, PsbK, PsbM, PsbO, PsbP, PsbQ, PsbR, PsbT, PsbW, and PsbZ, respectively. The letter P in a circle represents phosphate. The yellow lightning bolt represents light. Abbreviations: LHCII, light-harvesting complex II; OEC, oxygen-evolving complex; pD1, precursor D1; PSII, Photosystem II. For simplicity, only one name is shown for proteins with multiple names (e.g., “THF1” for THF1/PSB29).

The PSII repair cycle is a sequential process as well. The major steps in higher plants include: (1) high-light-induced phosphorylation, damage, and disassembly of the PSII-LHCII supercomplex and the PSII core dimer in grana stacks, (2) lateral migration of the PSII core monomer to stroma-exposed thylakoid membranes, (3–5) dephosphorylation, partial disassembly of the PSII core monomer, and degradation of photodamaged D1, (6) synthesis and reassembly of new D1, (7) re-incorporation of CP43, (8) re-attachment of OEC, (9) migration of the PSII core monomer back to grana stacks, and (10) dimerization into PSII core dimers and reformation of PSII-LHCII supercomplexes (Figure 2; Mulo et al., 2008; Järvi et al., 2015).

## Proteins that influence the assembly, stability, and repair of PSII

Assistance from a series of protein factors is required for the assembly, stability, and repair of PSII (Figures 1, 2; Mulo et al., 2008; Nickelsen and Rengstl, 2013; Järvi et al., 2015). The types of protein factors include: (1) components of thylakoid-protein-targeting complexes; (2) components of the chloroplast-vesicle-transport system, (3) PSII subunit-like proteins, e.g., PsbP-like and LHCP-like proteins, (4) atypical short-chain dehydrogenase/reductase (SDR) family proteins, (5) C-terminal D1 processing endopeptidases, (6) tetratricopeptide repeat (TPR) proteins, (7) thiol/disulfide-modulating proteins, (8) peptidylprolyl isomerases (PPIases), (9) protein kinases, (10) protein phosphatases, (11) FtsH (filamentation temperature sensitive protein H) proteases, (12) Deg (Degradation of periplasmic proteins) proteases, and (13) other auxiliary proteins with unique or unknown domain compositions (Table 1). As discussed below, many proteins that are involved in *de novo* PSII assembly also play roles in the repair and reassembly cycle of PSII.

**Table 1:** **Summary of proteins that influence the assembly, stability, and repair of PSII in Arabidopsis**.

**Name**	**Gene locus in *Synechocystis* sp. PCC 6803^a^**	**Gene locus in Arabidopsis^b^**	**Full-length Size (kDa)**	**Mature size (kDa)**	**Location**	**Protein classification**	**Function**	**References^c^**
cpSRP43	−	At2g47450	41	35	CS	Thylakoid protein targeting: cpSRP translocase	Insertion and assembly of PSII proteins such as D1, D2, and CP47, and LHCII subunits	Henry et al., 2007; Schunemann, 2007; Cline and Dabney-Smith, 2008; Albiniak et al., 2012
cpSRP54	slr1531	At5g03940	61	53	CS	Thylakoid protein targeting: cpSRP translocase	Insertion and assembly of PSII proteins such as D1, D2, and CP47, and LHCII subunits	Henry et al., 2007; Schunemann, 2007; Cline and Dabney-Smith, 2008; Albiniak et al., 2012; Walter et al., 2015
cpFtsY	slr2102	At2g45770	40	36	CS, TM	Thylakoid protein targeting: cpSRP translocase	Insertion and assembly of PSII proteins such as D1, D2, and CP47, and LHCII subunits	Henry et al., 2007; Schunemann, 2007; Cline and Dabney-Smith, 2008; Albiniak et al., 2012; Walter et al., 2015
ALB3	slr1471	At2g28800	50	45	TM	Thylakoid protein targeting: cpSRP translocase	Insertion and assembly of PSII proteins such as D1, D2, and CP47, and LHCII subunits	Pasch et al., 2005; Henry et al., 2007; Ma et al., 2007; Schunemann, 2007; Cline and Dabney-Smith, 2008; Walter et al., 2015
cpSecA1	sll0616	At4g01800	117	111	CS, TM	Thylakoid protein targeting: cpSec translocase	Insertion and assembly of PSII proteins such as PsbO	Cline and Theg, 2007; Schunemann, 2007; Cline and Dabney-Smith, 2008; Albiniak et al., 2012
cpSecA2		At1g21650	203	203	CS, TM	Thylakoid protein targeting: cpSec translocase	Insertion and assembly of PSII proteins such as PsbO	Cline and Theg, 2007; Schunemann, 2007; Cline and Dabney-Smith, 2008; Albiniak et al., 2012
cpSecE1	ssl3335	At4g14870	19	15	TM	Thylakoid protein targeting: cpSec translocase	Insertion and assembly of PSII proteins such as PsbO	Cline and Theg, 2007; Schunemann, 2007; Cline and Dabney-Smith, 2008; Albiniak et al., 2012
cpSecE2		At4g38490	17	12	TM	Thylakoid protein targeting: cpSec translocase	Insertion and assembly of PSII proteins such as PsbO	Cline and Theg, 2007; Schunemann, 2007; Cline and Dabney-Smith, 2008; Albiniak et al., 2012
cpSecY1	sll1814	At2g18710	59	51	TM	Thylakoid protein targeting: cpSec translocase	Insertion and assembly of PSII proteins such as D1 and PsbO	Cline and Theg, 2007; Henry et al., 2007; Schunemann, 2007; Cline and Dabney-Smith, 2008; Albiniak et al., 2012; Walter et al., 2015
cpSecY2		At2g31530	65	61	TM	Thylakoid protein targeting: cpSec translocase	Insertion and assembly of PSII proteins such as D1 and PsbO	Cline and Theg, 2007; Henry et al., 2007; Schunemann, 2007; Cline and Dabney-Smith, 2008; Albiniak et al., 2012; Walter et al., 2015
Tha4	slr1046	At5g28750	16	14	TM	Thylakoid protein targeting: cpTat translocase	Insertion and assembly of PSII proteins such as PsbP and PsbQ	Cline and Theg, 2007; Schunemann, 2007; Cline and Dabney-Smith, 2008; Albiniak et al., 2012
HCF106	slr1046	At5g52440	28	19	TM	Thylakoid protein targeting: cpTat translocase	Insertion and assembly of PSII proteins such as PsbP and PsbQ	Cline and Theg, 2007; Schunemann, 2007; Cline and Dabney-Smith, 2008; Albiniak et al., 2012
cpTatC	sll0194	At2g01110	37	34	TM	Thylakoid protein targeting: cpTat translocase	Insertion and assembly of PSII proteins such as PsbP and PsbQ	Cline and Theg, 2007; Schunemann, 2007; Cline and Dabney-Smith, 2008; Albiniak et al., 2012
CPRabA5e	−	At1g05810	29	24	CS, TM	Chloroplast vesicle transport	Transport of PSII proteins such as LHCB1, LHCB3, and CP47, to and from thylakoids	Karim et al., 2014; Karim and Aronsson, 2014
CYO1/SCO2	−	At3g19220	21	17	TM	Chloroplast vesicle transport; thiol/disulfide-modulating protein	Chloroplast and thylakoid biogenesis; folding and transport of cysteine-containing proteins such as CP43, CP47, and LHCB1; stability of PSI-LHCI and PSII-LHCII supercomplexes	Shimada et al., 2007; Albrecht et al., 2008; Muranaka et al., 2012; Tanz et al., 2012
THF1/PSB29	sll1414	At2g20890	34	27	CE, CS, TM	Chloroplast vesicle transport	Thylakoid biogenesis; dynamics of PSII-LHCII supercomplexes	Wang et al., 2004; Keren et al., 2005; Huang et al., 2006, 2013; Shi et al., 2012; Yamatani et al., 2013
TerC	−	At5g12130	42	34	TM	Chloroplast vesicle transport	Thylakoid biogenesis; co-translational insertion of PSII proteins such as D1, D2, and CP43	Kwon and Cho, 2008; Schneider et al., 2014
VIPP1	sll0617	At1g65260	36	32	ICE, TM	Chloroplast vesicle transport	Thylakoid biogenesis; transport and/or co-translational insertion of photosynthetic proteins such as D1	Kroll et al., 2001; Zhang et al., 2012; Zhang and Sakamoto, 2012; Walter et al., 2015
PPL1	sll1418	At3g55330	26	18	TL	PSII subunit-like: PsbP-like	PSII repair	Ishihara et al., 2007
ELIP1	−	At3g22840	20	16	TM	PSII subunit-like: LHCP-like	Binding of chlorophyll and/or stability of pigment-binding proteins and complexes during photoinhibition?	Hutin et al., 2003; Casazza et al., 2005; Heddad et al., 2006; Rossini et al., 2006
ELIP2	−	At4g14690	20	16	TM	PSII subunit-like: LHCP-like	Binding of chlorophyll and/or stability of pigment-binding proteins and complexes during photoinhibition?	Hutin et al., 2003; Casazza et al., 2005; Heddad et al., 2006; Rossini et al., 2006
SEP3.1/LIL3:1	−	At4g17600	29	25	TM	PSII subunit-like: LHCP-like	Anchoring geranylgeranyl reductase to thylakoid membranes; stabilizing LHCII	Tanaka et al., 2010; Takahashi et al., 2014; Lohscheider et al., 2015
SEP3.2/LIL3:2	−	At5g47110	29	24	TM	PSII subunit-like: LHCP-like	Anchoring geranylgeranyl reductase to thylakoid membranes; stabilizing LHCII	Tanaka et al., 2010; Takahashi et al., 2014; Lohscheider et al., 2015
HCF173	sll1218	At1g16720	66	57	CS, TM	atypical SDR	Translational initiation of the *psbA* mRNA	Schult et al., 2007; Link et al., 2012
HCF244	slr0399	At4g35250	44	38	TM	atypical SDR	Translational initiation of the *psbA* mRNA	Link et al., 2012
CtpA1	slr0008	At3g57680	56	47	TL	C-terminal processing peptidase	C-terminal processing of D1 under high light	Yamamoto et al., 2001; Yin et al., 2008
CtpA2		At4g17740	56	46	TL	C-terminal processing peptidase	C-terminal processing of D1	Yamamoto et al., 2001; Che et al., 2013
LPA1/PratA	slr2048	At1g02910	50	47	TM	TPR	Biogenesis and assembly of the D1 protein	Peng et al., 2006
MET1	−	At1g55480	37	30	TM	TPR	Supercomplex formation in PSII repair	Ishikawa et al., 2005a; Bhuiyan et al., 2015
LQY1	−	At1g75690	16	12	TL	Thiol/disulfide-modulating protein	Dissembly, folding, and/or reassembly of cysteine-containing PSII subunits and complexes and/or D1 synthesis and turnover, during PSII repair	Lu, 2011; Lu et al., 2011
PDI6/PDIL1-2	−	At1g77510	56	54	CS	Thiol/disulfide-modulating protein	Regulation of D1 synthesis	Houston et al., 2005; Wittenberg et al., 2014
TRX-M1	slr0623	At1g03680	20	14	CS	Thiol/disulfide-modulating protein	Assembly of CP47 into PSII	Cain et al., 2009; Wang et al., 2013
TRX-M2		At4g03520	20	13	CS	Thiol/disulfide-modulating protein	Assembly of CP47 into PSII	Cain et al., 2009; Wang et al., 2013
TRX-M4		At3g15360	21	13	CS	Thiol/disulfide-modulating protein	Assembly of CP47 into PSII	Cain et al., 2009; Wang et al., 2013
LTO1	slr0565	At4g35760	40	35	TM	Thiol/disulfide-modulating protein	Disulfide bond formation in PsbO	Feng et al., 2011; Karamoko et al., 2011; Lu et al., 2013
RBD1	slr2033	At1g54500	22	16	TM	Thiol/disulfide-modulating protein	PSII assembly and stability	Calderon et al., 2013
CYP20-3/ROC4	slr1251	At3g62030	34	23	CS	PPIase	Repair and reassembly of PSII under high light; redox regulation during stress acclimation	Lippuner et al., 1994; Cai et al., 2008; Dominguez-Solis et al., 2008; Park et al., 2013; Speiser et al., 2015
CYP38/TLP40	sll0408	At3g01480	48	38-40	TL	PPIase	Inhibiting dephosphorylation of PSII subunits during the PSII repair; conversion of PSII core monomers to PSII supercomplexes	Fulgosi et al., 1998; Vener et al., 1999; Rokka et al., 2000; Fu et al., 2007; Sirpiö et al., 2008; Vasudevan et al., 2012
FKBP20-2	slr1761	At3g60370	27	20	TM, TL	PPIase	Formation of PSII-LHCII supercomplexes under normal and high light	Lima et al., 2006
STN7	−	At1g68830	63	59	TM	Protein kinase	Phosphorylation of LHCII; phosphorylation of D1, D2, CP43, and PsbH under low light	Bellafiore et al., 2005; Bonardi et al., 2005; Tikkanen et al., 2008; Pesaresi et al., 2011; Tikkanen and Aro, 2012
STN8	−	At5g01920	55	50	TM	Protein kinase	Phosphorylation of D1, D2, CP43, and PsbH	Bonardi et al., 2005; Tikkanen et al., 2008; Pesaresi et al., 2011; Tikkanen and Aro, 2012; Nath et al., 2013
PBCP	−	At2g30170	32	30	CS, TM	Protein phosphatase	Dephosphorylation of D1, D2, CP43, and PsbH	Samol et al., 2012
TLP18.3	sll1390	At1g54780	31	18	TL, TM	Protein phosphatase	D1 degradation and PSII dimerization; dephosphorylation of PSII core proteins (e.g., D1 and D2)	Sirpiö et al., 2007; Wu et al., 2011
PPH1/TAP38	−	At4g27800	43	41	TM	Protein phosphatase	Dephosphorylation of LHCII	Pribil et al., 2010; Shapiguzov et al., 2010; Pesaresi et al., 2011
FtsH1	slr1390, slr0228, slr1604, sll1463^d^	At1g50250	77	71	TM	FtsH protease	Degradation of photodamaged D1	Sakamoto et al., 2003; Yu et al., 2004, 2005; Zaltsman et al., 2005b
FtsH2/VAR2		At2g30950	74	69	TM	FtsH protease	Chloroplast biogenesis; thylakoid formation; degradation of photodamaged D1	Bailey et al., 2002; Sakamoto et al., 2002, 2003; Yu et al., 2004, 2005; Zaltsman et al., 2005a,b; Kato et al., 2007, 2009, 2012; Wagner et al., 2011
FtsH5/VAR1		At5g42270	75	69	TM	FtsH protease	Chloroplast biogenesis; thylakoid formation; degradation of photodamaged D1	Sakamoto et al., 2002, 2003; Yu et al., 2004, 2005; Zaltsman et al., 2005b; Kato et al., 2009; Wagner et al., 2011
FtsH6		At5g15250	77	69	TM	FtsH protease	Degradation of LHCII during high-light acclimation and senescence?	Sakamoto et al., 2003; Yu et al., 2004; Zelisko et al., 2005; Wagner et al., 2011
FtsH8		At1g06430	73	69	TM	FtsH protease	Degradation of photodamaged D1	Sakamoto et al., 2003; Yu et al., 2004, 2005; Zaltsman et al., 2005b; Wagner et al., 2011
FtsH11		At5g53170	89	82	C(TM?), IMM	FtsH protease	Thermoprotection of the photosynthetic apparatus	Sakamoto et al., 2003; Yu et al., 2004; Urantowka et al., 2005; Chen et al., 2006b; Wagner et al., 2011
Deg1	slr1204, sll1679, sll1427^e^	At3g27925	47	42	TL, TM,	Deg protease	Degradation of plastocyanin and PsbO, and photodamaged D1; integration of newly synthesized PSII subunits such as D1, D2, CP43, and CP47, into PSII complexes	Chassin et al., 2002; Huesgen et al., 2005; Kapri-Pardes et al., 2007; Sun et al., 2010b; Schuhmann and Adamska, 2012
Deg2		At2g47940	67	59	CS, TM	Deg protease	Stress-induced degradation of LHCB6; a minor protease in *in vivo* degradation of photodamaged D1	Haubühl et al., 2001; Huesgen et al., 2005, 2006; Luciński et al., 2011b; Schuhmann and Adamska, 2012
Deg5		At4g18370	35	32	TL, TM	Deg protease	Degradation of photodamaged D1; wound-induced degradation of PsbF	Huesgen et al., 2005; Sun et al., 2007; Luciński et al., 2011a; Kato et al., 2012; Schuhmann and Adamska, 2012
Deg7		At3g03380	120	120?	CS, TM	Deg protease	Degradation of photodamaged D1, D2, CP43, and CP47	Huesgen et al., 2005; Sun et al., 2010a; Schuhmann et al., 2011; Schuhmann and Adamska, 2012
Deg8		At5g39830	47	45	TL, TM	Deg protease	Degradation of photodamaged D1	Huesgen et al., 2005; Sun et al., 2007; Kato et al., 2012; Schuhmann and Adamska, 2012
HCF243	−	At3g15095	76	67	TM	Other^f^	Biogenesis, C-terminal processing, and assembly of D1; possibly biogenesis of D2	Zhang et al., 2011
PSB27-H1	slr1645	At1g03600	19	12	TL, TM	Other	C-terminal processing of D1 during PSII repair?	Chen et al., 2006a; Wei et al., 2010; Dietzel et al., 2011; Shi et al., 2012; Mabbitt et al., 2014
PSB27-H2/LPA19		At1g05385	22	15	TL, TM	Other	C-terminal processing during *de novo* PSII assembly	Wei et al., 2010; Shi et al., 2012; Mabbitt et al., 2014
HCF136	slr2034	At5g23120	44	38	TL	Other	Assembly of PSII reaction-center complexes such as RC, RC47a, and RC47b	Meurer et al., 1998; Plücken et al., 2002; Mabbitt et al., 2014
PAM68	sll0933	At4g19100	24	20	TM	Other	Conversion of PSII minimal reaction-center complexes into larger PSII assembly intermediates; C-terminal processing of D1	Armbruster et al., 2010
PsbN/PBF1	smr0009	AtCg00700	4.7	4.7	TM	Other	Assembly of PSII minimal reaction-center complexes; regulation of PSII core and antenna protein phosphorylation	Krech et al., 2013; Torabi et al., 2014
PSB28	sll1398	At4g28660	22	14	TL	Other	Biogenesis of chlorophyll-binding proteins such as CP47, PsaA, and PsaB	Jung et al., 2008; Shi et al., 2012; Mabbitt et al., 2014
LPA2	−	At5g51545	20	~20	TM	Other	Synthesis and assembly of CP43	Ma et al., 2007; Cai et al., 2010
LPA3	−	At1g73060	40	34	CS, TM	Other	Synthesis and assembly of CP43	Cai et al., 2010
PSB33	−	At1g71500	32	25	TM	Other	Association of LHCII with PSII	Fristedt et al., 2015
HHL1	−	At1g67700	26	18	TM	Other	Reassembly of PSII core monomers and PSII-LHCII supercomplexes during PSII repair	Jin et al., 2014
MPH1	−	At5g07020	24	20	TM	Other	Assembly and/or stability of PSII core monomers and higher order PSII complexes under high light	Liu and Last, 2015a,b

C, chloroplast; CE, chloroplast envelope; CS, chloroplast stroma; ICE, inner chloroplast envelope; IMM, inner mitochondria membranes; TL, thylakoid lumen; TM, thylakoid membranes.

a The gene loci in Synechocystis sp. PCC 6803 are included in this table only to distinguish between factors that are conserved in cyanobacteria and land plants and factors that are found in land plants but not in cyanobacteria. The hyphen indicates that the corresponding factor is either absent or not yet found in Synechocystis sp. PCC 6803.

b This article focuses on the identification and roles of PSII assembly, stability, and repair factors in Arabidopsis; therefore the gene loci for factors in other land plants are not listed in this table.

c Detailed descriptions of factors involved in the assembly, stability, and repair of PSII in cyanobacteria can be found in Nickelsen and Rengstl (2013). Therefore, references for factors in cyanobacteria are not listed in this table.

d The Synechocystis sp. PCC 6803 genome contains four FtsH genes. Because there is no straightforward one-to-one correspondence between the 12 Arabidopsis FtsH genes and four cyanobacterial FtsH genes, the four cyanobacterial FtsH loci are listed together.

e The Synechocystis sp. PCC 6803 genome contains three Deg genes. Because there is no straightforward one-to-one correspondence between the 16 Arabidopsis Deg genes and three cyanobacterial Deg genes, the three cyanobacterial Deg loci are listed together.

f Factors that do not fall in the 12 well-defined classifications (see Section Proteins that influence the assembly, stability, and repair of PSII) are classified as “other proteins” that influence the assembly, stability, and repair of PSII.

Some of these factors, such as components of the thylakoid-protein-targeting complexes and the chloroplast-vesicle-transport system, are not specific for the assembly, stability, or repair of PSII. However, because thylakoid protein targeting and chloroplast vesicle transport are essential for translocation and accumulation of thylakoid membrane/lumen proteins, and because most of PSII subunits and assembly, stability, and repair factors are thylakoid membrane/lumen proteins, these two types of protein factors are included in this article.

### Components of thylakoid-protein-targeting complexes

Four thylakoid transport and integration pathways have been identified to date (Cline and Dabney-Smith, 2008; Albiniak et al., 2012). The cpSRP pathway and an unusual pathway that requires none of the known targeting apparatus are responsible for translocating thylakoid membrane proteins. LHCPs are translocated via the cpSRP pathway that requires the action of cpSRP43 (chloroplast Signal Recognition Particle protein of 43 kDa), cpSRP54 (cpSRP protein of 54 kDa), cpFtsY (chloroplast filamentation temperature sensitive protein Y), and ALB3 (Albino3) (Figures 1, 2). ALB3 was found to interact with PSII subunits D1, D2, and CP47 (Ossenbühl et al., 2004; Pasch et al., 2005; Göhre et al., 2006), consistent with the function of the cpSRP pathway in translocating PSII proteins such as D1, D2, and CP47 (Figures 1, 2). Most of the remaining thylakoid membrane proteins are inserted by the unusual pathway that requires none of the known targeting apparatus. The cpSecA-cpSecYE (cpSec means chloroplast secretory) pathway and the chloroplast twin-arginine translocation (cpTat) pathway are responsible for translocation of lumenal proteins. In Arabidopsis, each cpSec component is encoded by two genes (Table 1). The cpTat pathway has three components: Thylakoid Assembly 4 (THA4), High Chlorophyll Fluorescence 106 (HCF106), and cpTat protein C (cpTatC) (Albiniak et al., 2012). PsbO and plastocyanin are substrates of the cpSec pathway while PsbP and PsbQ are substrates of the cpTat pathway (Figures 1, 2; Albiniak et al., 2012). Like their bacterial counterparts, components of the cpSRP and cpSec pathways may act in a modular fashion (Cline and Theg, 2007; Henry et al., 2007). For instance, cpSRP54 was identified in co-translational D1 insertion intermediates, along with cpSecY (chloroplast secretory translocase Y) and chloroplast ribosomes (Cline and Theg, 2007; Henry et al., 2007). Consistent with this finding, Walter et al. (2015) reported that cpSecY forms a complex with VIPP1 (Vesicle-Inducing Protein in Plastids 1) and cpSec components ALB3, cpFtsY, and cpSRP54 during co-translational integration of D1 (Figures 1, 2). How different thylakoid membrane and lumenal proteins, including components of PSII complexes, are transported and integrated into thylakoids by the above-mentioned thylakoid-protein-targeting complexes can be found in a number of reviews (Schunemann, 2007; Cline and Dabney-Smith, 2008; Albiniak et al., 2012).

### Components of the putative chloroplast-vesicle-transport system

PSII is located in the thylakoid membranes of oxygenic photosynthetic organisms; therefore thylakoid membrane biogenesis is essential to PSII assembly (Nickelsen and Zerges, 2013; Rast et al., 2015). It has been suggested that thylakoids could develop from invaginations of the inner envelope membrane via a vesicle-based transfer process and these vesicles are thought as a method of transporting lipids and proteins to and from thylakoids (Hoober et al., 1991; Westphal et al., 2001, 2003; Charuvi et al., 2012; Karim and Aronsson, 2014; Rast et al., 2015). Chloroplast vesicles are typically not observed when vesicular transport from the inner chloroplast envelope to thylakoids is continuous at ambient temperature (Morré et al., 1991). When vesicular transport is blocked by low temperature, chloroplast vesicles accumulate (Morré et al., 1991). A number of proteins have been implicated as part of the chloroplast-vesicle-transport system, including CPRabA5e (chloroplast Rab GTPase A5e), CYO1/SCO2 (Shiyou 1/Snowy Cotyledon 2), THF1/PSB29 (Thylakoid Formation 1/PSII protein 29), TerC (Tellurite-resistance protein C), and VIPP1 (Karim and Aronsson, 2014; Rast et al., 2015).

CPRabA5e is a small Rab GTPase targeted to the chloroplast stroma and thylakoid membranes (Karim et al., 2014). Transfer DNA (T-DNA) insertions in the *CPRabA5e* gene cause a reduced amount of grana thylakoids in Arabidopsis leaves (Karim et al., 2014). After pre-incubation at 4°C, the *cprabA5e* mutants have larger plastoglobules and an increased number of small vesicles, compared to the wild type. CPRabA5e was found to interact with a number of photosynthetic proteins, including PSI subunits H2 and K (PsaH2 and PsaK; Psa stands for Photosystem I), the PSII core subunit CP47, and LHCB proteins LHCB1 and LHCB3 (Karim et al., 2014). These findings led to the hypothesis that CPRabA5e is involved in transport of photosynthetic proteins, such as LHCB1, LHCB3, and CP47 (Figures 1, 2), to developing thylakoids in young chloroplasts via vesicles (Karim et al., 2014).

CYO1/SCO2 is a zinc-finger-domain-containing thylakoid membrane protein with protein disulfide isomerase (PDI) activity (Shimada et al., 2007; Albrecht et al., 2008; Muranaka et al., 2012; Tanz et al., 2012). A loss-of-function mutation in the *CYO1/SCO2* gene results in globularly or normally shaped plastids with very big vesicles in Arabidopsis cotyledons (Tanz et al., 2012). A closer look at normally shaped chloroplasts from mutant cotyledons showed that small vesicles emerged from the inner chloroplast envelope, even at ambient temperature. These vesicles were not observed in chloroplasts from wild-type cotyledons used in the same study. These observations suggest that vesicular transport from the inner chloroplast envelope to developing thylakoids is blocked in the cotyledons of mutant seedlings and that the chloroplast-vesicle-transport system is important for thylakoid biogenesis rather than damage or programmed degradation of the thylakoid membrane system. CYO1/SCO2 was found to interact with PSI core subunits PsaA and PsaB, PSII core subunits CP43 and CP47, and LHCB1 (Muranaka et al., 2012; Tanz et al., 2012) and to co-migrate with Photosystem I-light-harvesting complex I (PSI-LHCI) and PSII-LHCII supercomplexes in blue native-polyacrylamide gel electrophoresis (BN-PAGE) (Shimada et al., 2007). Therefore, it is conceivable that CYO1/SCO2 participates in transport of photosynthetic proteins, such as CP43, CP47, and LHCB1 (Figures 1, 2), to and from thylakoids via chloroplast vesicles in cotyledons (Tanz et al., 2012). The association of CYO1/SCO2 with PSI-LHCI and PSII-LHCII supercomplexes also begs the question whether this protein has a role in stabilizing these supercomplexes (Figures 1, 2).

THF1/PSB29 is a coiled-coil-domain-containing protein targeted to the chloroplast envelope, chloroplast stroma, and thylakoid membranes (Wang et al., 2004; Huang et al., 2006). The *thf1/psb29* knockout mutant of Arabidopsis has a variegated phenotype in cotyledons and true leaves and the chloroplasts in the yellow sectors of *thf1/psb29* leaves lack normal thylakoids and accumulate chloroplast vesicles (Wang et al., 2004; Keren et al., 2005; Zhang et al., 2009). These data led to the hypothesis that THF1/PSB29 is required for organizing chloroplast vesicles into mature thylakoids (Wang et al., 2004). THF1/PSB29 was thought to be involved in maintaining levels of FtsHs in plants because D1, a substrate of FtsHs, was found to be more stable in the *thf1/psb29* mutant than in the wild type (Zhang et al., 2009). However, it was later found that the stay-green phenotype of the *thf1/psb29* mutant is not due to reduced FtsH protease activity, because *ftsh2/var2* (*var2* stands for *yellow variegated 2*) leaves turn yellow much faster than wild-type and *thf1/psb29* leaves during dark-induced senescence (Huang et al., 2013). THF1/PSB29 was found to interact with all six LHCB proteins (Huang et al., 2013); therefore it is possible that LHCB proteins are transported to thylakoid membranes via direct interaction with THF1/PSB29 (Figures 1, 2). In addition, it was proposed that THF1/PSB29 regulates the dynamics of PSII-LHCII supercomplexes during high-light stress and leaf senescence (Figure 2; Huang et al., 2013; Yamatani et al., 2013). The THF1/PSB29-deficient mutants of Arabidopsis and rice (*Oryza sativa*) also have a stay-green phenotype in pathogen-infected and dark-induced senescent leaves (Huang et al., 2013; Yamatani et al., 2013). In dark- and high-light-treated *thf1/psb29* Arabidopsis leaves, PSII-LHCII supercomplexes are highly unstable but a type of PSII-LHCII megacomplexes is retained (Huang et al., 2013). Consistent with a role in regulating the dynamics of PSII-LHCII supercomplexes, THF1/PSB29 was found to co-migrate with trimeric and monomeric LHCII in BN-PAGE (Huang et al., 2013).

TerC is an integral thylakoid membrane protein with eight transmembrane helices (Kwon and Cho, 2008; Schneider et al., 2014). T-DNA insertions in the *TerC* gene caused a pigment-deficient and seedling-lethal phenotype in Arabidopsis (Kwon and Cho, 2008; Schneider et al., 2014). This is accompanied with a substantial reduction or complete loss of thylakoid membranes and over-accumulation of chloroplast vesicles. Therefore, TerC was considered to be invloved in thylakoid biogenesis and vesicle transport (Kwon and Cho, 2008). To further analyze the function of TerC, Schneider et al. (2014) generated an artifical microRNA-based knockdown allele *amiR-TerC* in Arabidopsis and found that the severe phenotype of the T-DNA mutants is likely due to the subsantaially reduced rates of synthesis and insertion of PSII proteins. In line with these observations, TerC was found to interact with PSII proteins D1, D2, and CP43 as well as PSII assembly factors ALB3, LPA1/PratA (Low PSII Accumulation 1/Processing-associated tetratricopeptide repeat protein A), LPA2 (Low PSII Accumulation 2), and PAM68 (Photosynthesis Affected Mutant 68) (Schneider et al., 2014). Taken together, it is reasonable to propose that TerC is also invloved in co-translational insertion of PSII proteins (e.g., D1, D2, and CP43) into thylakoid membranes, in colaboration with other PSII assembly factors (Figures 1, 2; Schneider et al., 2014).

VIPP1 is homologous to the phage shock protein A in *Escherichia coli*, which is induced under various stress environments (Karim and Aronsson, 2014). VIPP1 is targeted to the inner chloroplast envelope and thylakoid membranes (Kroll et al., 2001). The *vipp1* knockdown and knockout mutants of Arabidopsis are pigment-deficient and semi-lethal (i.e., unable to grow photoautotrophically) (Kroll et al., 2001; Zhang et al., 2012; Zhang and Sakamoto, 2012). Chloroplasts from the *vipp1* knockout and knockdown mutants are defective in thylakoid membrane formation and vesicle budding from inner envelope membranes. In addition, *vipp1* mutant chloroplasts are swollen due to damage in the chloroplast envelope and increases in the osmotic pressure in the chloroplast stroma (Zhang et al., 2012; Zhang and Sakamoto, 2012). VIPP1 was recently found in co-translational D1 insertion intermediates isolated from thylakoid membranes of pea (*Pisum sativum*) leaves, along with cpSecY, ALB3, cpFtsY, and cpSRP54 (Walter et al., 2015). Therefore, VIPP1 was proposed as a multifunctional protein that is involved in chloroplast vesicular transport, thylakoid biogenesis, and co-translational insertion of photosynthetic proteins (Figure 1).

### PSII subunit-like proteins, e.g., PsbP-like and LHCP-like proteins

Five PSII subunit-like proteins, including one PsbP-like protein and four LHCP-like proteins, have been implicated to be involved in the assembly, stability, and/or repair of PSII complexes or subunits. PsbP-like proteins are in the same family as PsbP proteins (Bricker et al., 2013; Ifuku, 2014). Unlike PsbP proteins, whose primary function is water splitting and oxygen evolution, PsbP-like proteins are not part of the OEC. Arabidopsis has two PsbP-like proteins: PPL1 and PPL2 (Table 1). A T-DNA insertion in the *PPL1* gene led to increased sensitivity to high light and delayed recovery after photoinhibition (Ishihara et al., 2007). These data suggest that PPL1 is required for efficient repair of photodamaged PSII. Although PPL1 has not been shown to be associated with PSII in higher plants, its cyanobacterial homolog cyanoP (ssl1418) has been shown to be loosely associated with PSII and possess the same beta-sandwich fold and a well-conserved zinc-binding site as PsbP in higher plants (Ishikawa et al., 2005b; Summerfield et al., 2005; Michoux et al., 2010, 2014; Jackson et al., 2012). Further studies are needed to investigate the precise function of PPL1 in PSII repair. Unlike PPL1, PPL2 is required for the accumulation of chloroplast NAD(P)H dehydrogenase complex (Ishihara et al., 2007); therefore the function of PPL2 is not discussed in this review.

LHCP-like proteins are in the same superfamily as LHCPs (Heddad et al., 2012). Unlike LHCPs, whose primary function is light-harvesting, most LHCP-like proteins are involved in chlorophyll- and carotenoid-binding, assembly and stability of chlorophyll-protein complexes, and/or photoprotection. LHCP-like proteins can be classified into three subfamilies: three-helix, early light-induced proteins (ELIPs), two-helix, stress-enhanced proteins (SEPs), and one-helix, high-light-induced, small chlorophyll-binding-like proteins (OHPs/HLIPs/SCPs). ELIPs are restricted to green algae and land plants, SEPs are ubiquitously present in photosynthetic eukaryotes, OHP1 exists in cyanophages, cyanobacteria, and photosynthetic eukaryotes, and OHP2 exists in eukaryotes. The Arabidopsis genome encodes two ELIPs, six SEPs, and two OHPs (Heddad et al., 2012).

ELIPs appear to be associated with PSII under standard conditions in pea plants; they become associated with monomeric and trimeric LHCII under high light in Arabidopsis (Adamska and Kloppstech, 1991; Heddad et al., 2006). Heddad et al. (2006) showed that the relative amounts of ELIP transcripts and proteins increase as the light intensity increases. It was therefore proposed that ELIPs may be involved in photoprotection by binding free chlorophyll released during degradation of pigment-binding proteins or by stabilizing the assembly of pigment-binding proteins during photoinhibition (Adamska and Kloppstech, 1991; Hutin et al., 2003). The potential function of ELIPs in stabilizing LHCII and PSII-LHCII supercomplexes under light stress is included in Figure 2. However, *elip1* and *elip2* single knockout mutants and the *elip1 elip2* double knockout mutant have a similar phenotype and light sensitivity as the wild-type Arabidopsis plants (Casazza et al., 2005; Rossini et al., 2006). Additional studies are needed to dissect the exact functions of ELIP proteins.

Among the six SEPs in Arabidopsis, the functions of SEP3.1/LIL3:1 (SEP stands for stress-enhanced protein; LIL stands for light-harvesting-like protein) and SEP3.2/LIL3:2 have been extensively studied (Tanaka et al., 2010; Takahashi et al., 2014; Lohscheider et al., 2015; Mork-Jansson et al., 2015). The anti-sense *sep3.1/lil3:1* mutant and the *sep3.1/lil3:1 sep3.2/lil3:2* double knockout mutant are deficient in chlorophyll and α-tocopherol biosynthesis (Tanaka et al., 2010; Lohscheider et al., 2015). The deficiency is due to a substantial reduction in the amount of chlorophyll and α-tocopherol biosynthetic enzyme geranylgeranyl reductase. In line with these findings, SEP3.1/LIL3:1 and SEP3.2/LIL3:2 were found to interact with geranylgeranyl reductase and their transmembrane domain was found to be important for the interaction (Tanaka et al., 2010; Takahashi et al., 2014). Therefore, SEP3.1/LIL3:1 and SEP3.2/LIL3:2 were proposed to be involved in chlorophyll and tocopherol biosynthesis by anchoring and stabilizing geranylgeranyl reductase to thylakoid membranes. In addition, SEP3.1/LIL3:1 and SEP3.2/LIL3:2 were found to accumulate with increasing light irradiance and they are associated with subcomplexes of LHCII (Lohscheider et al., 2015). Thus, it is also possible that SEP3.1/LIL3:1 and SEP3.2/LIL3:2 may function in stabilizing LHCII (Figures 1, 2).

The *OHP* genes have an expression pattern similar to the *ELIP* and *SEP* genes, whose expression is up-regulated upon high light (Mulo et al., 2008; Heddad et al., 2012). The function of OHP1 is not yet known, but OHP2 was showed to be associated with PSI under low or high light and was therefore proposed to play a role in photoprotection of PSI (Andersson et al., 2003). Recently, a cyanobacterial OHP family protein was found to bind chlorophyll *a* and β-carotene and possess an energy-dissipative conformation, suggesting that OHP family proteins may have a photoprotective role (Staleva et al., 2015).

### Atypical SDR family proteins

Classic SDR family proteins have an intact cofactor-binding site (TGXXGXXG) and an intact catalytic tetrad (NSYK), which are required for their SDR activity (Persson et al., 2009). Unlike classic SDR family proteins, atypical SDR family proteins have no known enzyme activity because they have an altered glycine-rich cofactor-binding site and partially or completely lack the signature catalytic tetrad (Link et al., 2012). Two atypical SDR family proteins have been found to be important for PSII: HCF173 (High Chlorophyll Fluorescence 173) and HCF244 (High Chlorophyll Fluorescence 244) (Schult et al., 2007; Link et al., 2012; Chidgey et al., 2014; Knoppová et al., 2014). Compared to HCF244, HCF173 is ~200 amino acids longer and its SDR domain is fragmented into two regions. HCF173 and HCF244 have the same subcellular localization: they are both predominantly associated with chloroplast membranes, with a small fraction located in the chloroplast stroma. Loss-of-function mutations in the *HCF173* or *HCF244* gene result in similar defects in Arabidopsis: a drastic reduction in D1 synthesis, inability to accumulate PSII subunits, substantial decreases in PSII activity, and a complete loss of photoautotrophy (Schult et al., 2007; Link et al., 2012). The *hcf173* and *hcf244* single mutants are able to grow on sucrose-supplemented media but they are pale green and much smaller than the wild type (Link et al., 2012). Polysome association experiments demonstrated that these defects are caused by reduced translation initiation of the *psbA* transcript (Schult et al., 2007; Link et al., 2012). The decrease in translation initiation is accompanied by a reduction in *psbA* mRNA stability. The *hcf173 hcf244* double mutant grown on sucrose-supplemented media is smaller than the single mutants, suggesting that simultaneous loss of HCF173 and HCF244 has an additive effect (Link et al., 2012). The function of HCF173 and HCF244 in translation initiation of the *psbA* transcript is included in Figures 1, 2. Some SDR family proteins, such as dihydrolipoamide acetyltransferases, glyceraldehyde-3-phosphate dehydrogenase, and lactate dehydrogenase, have evolved the capacity to bind RNA (Hentze, 1994; Nagy et al., 2000; Pioli et al., 2002; Bohne et al., 2013). Therefore, it is possible that HCF173 and HCF244 may act as RNA-binding proteins and facilitate translation initiation of the *psbA* mRNA (Link et al., 2012).

### C-terminal processing peptidases

The PSII reaction-center protein D1 is often synthesized in the precursor form (pD1), with a C-terminal extension of 8–16 amino acids (Nixon et al., 1992; Anbudurai et al., 1994; Liao et al., 2000). In plants, this C-terminal extension is cleaved in a single step by C-terminal processing peptidase A (CtpA) at an early step of *de novo* PSII assembly or at the reassembly step of PSII repair (Figures 1, 2). CtpAs are serine endopeptidases with a serine/lysine catalytic dyad (Anbudurai et al., 1994; Liao et al., 2000; Yamamoto et al., 2001). Recombinant spinach (*Spinacia oleracea*) CtpA exhibited efficient proteolytic activity toward thylakoid membrane-embedded pD1 (Yamamoto et al., 2001). Arabidopsis has three CtpAs in the thylakoid lumen and the functions of CtpA1 and CtpA2 have been studied (Yin et al., 2008; Che et al., 2013).

Under normal growth conditions, T-DNA insertions in the Arabidopsis *CtpA1* gene do not cause changes to plant growth and morphology, PSII activity, or thylakoid membrane complex formation (Yin et al., 2008). Under high light, the *ctpA1* mutant displays retarded growth, accelerated D1 turnover, as well as increased photosensitivity and delayed recovery of PSII activity. Therefore, CtpA1 was proposed to be involved in D1 protein C-terminal processing in the PSII repair cycle (Figure 2).

Unlike the *ctpA1* mutant, the T-DNA mutant of the Arabidopsis *CtpA2* gene is lethal under normal light but is viable in sucrose-supplemented media under low light (Che et al., 2013). The viable *ctpA2* mutant displays a complete loss of the mature D1 protein, reduced levels of other PSII core proteins, a severely decreased level of PSII supercomplexes, and a substantial reduction or complete loss of PSII activity. pD1 and other PSII subunits in the viable *ctpA2* mutant are present in PSII monomers and PSII dimers but absent in PSII supercomplexes. These data suggest that CtpA2 is indispensible for C-terminal processing of D1 (Figure 1), which in itself is essential for *de novo* PSII assembly. A weak allele expressing ~2% of the wild-type level of CtpA2 appears to be normal under normal light but displays stunted growth and over-accumulation of pD1 under elevated light (Che et al., 2013). These data suggest that CtpA2 is also involved in C-terminal processing of D1 during high-light-induced PSII repair (Figure 2).

### TPR proteins

The TPR is a 34-amino acid repeated motif that ubiquitously exits among all organisms (Ishikawa et al., 2005a). Two TPR proteins have been found to be involved in PSII assembly and/or repair: LPA1/PratA and MET1 (Mesophyll-Enriched Thylakoid protein 1) (Ishikawa et al., 2005a; Peng et al., 2006; Bhuiyan et al., 2015).

LPA1/PratA is an intrinsic thylakoid membrane protein with two tandem TPR motifs and a double-pass transmembrane domain (Klinkert et al., 2004; Peng et al., 2006). T-DNA insertions in the Arabidopsis *LPA1/PratA* gene result in reduced growth, pale-green leaves, reduced PSII activity, reduced amounts of PSII proteins, reduced synthesis of D1 and D2, increased turnover of PSII core subunits D1, D2, CP43, and CP47, and inefficient assembly of PSII (Peng et al., 2006). The transcript levels of genes encoding PSII core subunits are unchanged in the mutants. LPA1/PratA was found to directly interact with D1 in a split-ubiquitin yeast-two-hybrid assay. These data suggest that LPA1/PratA has a role in biogenesis and assembly of D1 (Figures 1, 2). Consistent with this hypothesis, LPA1/PratA was identified in thylakoid-membrane-associated ribosome nascent chain fractions (Peng et al., 2006).

MET1 has an N-terminal PDZ domain and a C-terminal TPR motif, which are conserved across green algae and land plants (Ishikawa et al., 2005a; Bhuiyan et al., 2015). MET1 is peripherally attached to thylakoid membranes on the stromal side and it is enriched in stroma lamellae (Bhuiyan et al., 2015). T-DNA insertions in the Arabidopsis *MET1* gene do not cause obvious changes to the accumulation and assembly state of the photosynthetic apparatus under normal light (Bhuiyan et al., 2015). Under fluctuating light, the *met1* mutants demonstrate reduced growth, decreased PSII efficiency, a near-complete loss of PSII-LHCII supercomplexes, and increased amounts of unassembled CP43. Loss of MET1 also causes increased photosensitivity of PSII activity and an accelerated rate of D1 turnover under high light. MET1 was found to co-migrate with a series of PSII subcomplexes, such as PSII dimers, PSII core monomers, CP43-less PSII monomers, and PSII reaction-center complexes (i.e., RC, RC47a, and RC47b), in BN-PAGE. Therefore, MET1 was proposed to be involved in supercomplex formation during PSII repair (Figure 2). In line with this hypothesis, MET1 was found to interact with the stromal loops of PSII core subunits CP43 and CP47 (Bhuiyan et al., 2015).

### Thiol/disulfide-modulating proteins

Thiol/disulfide modulation is important for regulating photosynthetic processes (Järvi et al., 2013; Karamoko et al., 2013). Three types of thiol/disulfide-modulating proteins have been found to be involved in the assembly, stability, function, and repair of PSII: protein disulfide isomerases (PDIases), protein disulfide reducing proteins, and protein thiol oxidizing proteins.

As mentioned above, thylakoid membrane protein CYO1/SCO2 was found to play a role in chloroplast and thylakoid biogenesis and vesicular transport of photosynthetic proteins to developing thylakoids in cotyledons (Shimada et al., 2007; Albrecht et al., 2008; Tanz et al., 2012). CYO1/SCO2 has a C_4_-type zinc-finger domain with two conserved CXXCXGXG repeats, the signature domain for PDIase activity (Shimada et al., 2007). Recombinant CYO1/SCO2 is able to catalyze reduction of protein disulfide bonds and oxidative renaturation of reduced and denatured protein substrates, indicating that CYO1/SCO2 is a PDIase (Shimada et al., 2007). CYO1/SCO2 was found to interact with PsaA, PsaB, CP47, CP43, and LHCB1 (Muranaka et al., 2012; Tanz et al., 2012). These five CYO1/SCO2-interacting proteins contain cysteine in the hydrophobic region(s). Therefore, CYO1/SCO2 may also participate in folding of cysteine-containing PSI and PSII subunits (Figures 1, 2), by forming transient disulfide bonds with its protein substrates via the CXXC motif (Jessop et al., 2007; Feige and Hendershot, 2011).

LQY1 (Low Quantum Yield of PSII 1) is another thylakoid membrane protein with PDIase activity. Full-length LQY1 has a chloroplast transit peptide, a transmembrane domain, and a C-terminal C_4_-type zinc-finger domain with four conserved CXXCXGXG repeats (Lu et al., 2011). Recombinant LQY1 is able to catalyze oxidative renaturation of reduced and denatured protein substrates and reductive renaturation of oxidized protein substrates. T-DNA insertions in the Arabidopsis *LQY1* gene cause reduced efficiency of PSII photochemistry, increased sensitivity to high light, and increased accumulation of reactive oxygen species under high light. The *lqy1* mutants were found to accumulate fewer PSII-LHCII supercomplexes and have altered rates of high-light-induced D1 turnover and re-synthesis. The *lqy1* mutant phenotype can be suppressed by complementation of *lqy1* mutants with the wild-type *LQY1* gene (Lu, 2011). LQY1 is associated with the PSII core monomer and the CP43-less PSII monomer (a marker for ongoing PSII repair and reassembly) and it is most abundant in stroma-exposed thylakoid membranes, where important steps of PSII repair occurs (Lu et al., 2011). Under high light, LQY1 associated with PSII monomers increases at the expense of free LQY1 and LQY1 associated with smaller PSII complexes. Immunoprecipitation analysis showed that LQY1 interacts with PSII core subunits CP47 and CP43, which contain three and four conserved cysteine residuals, respectively. Therefore, it was proposed that LQY1 is involved in PSII repair (Lu, 2011; Lu et al., 2011). It is possible that LQY1 participates in dissembly, folding, and/or reassembly of cysteine-containing PSII subunits and complexes and/or regulates D1 synthesis and turnover during PSII repair (Figure 2). These hypotheses require further investigation.

PDI6/PDIL1-2 (Protein Disulfide Isomerase 6/Protein Disulfide Isomerase-Like 1-2) contains two redox-active thioredoxin domains (with the WCGHC active site), two redox-inactive thioredoxin-like domains, and a C-terminal endoplasmic reticulum retention signal KDEL (Houston et al., 2005; Wittenberg et al., 2014). PDI6/PDIL1-2 is dual-targeted to chloroplasts and the endoplasmic reticulum; chloroplast–targeted PDI6 is located in the stroma (Wittenberg et al., 2014). Similar to CYO1/SCO2 and LQY1, recombinant PDI6/PDIL1-2 is capable of catalyzing oxidative renaturation of reduced and denatured protein substrates. Compared to wild-type Arabidopsis, the *pdi6-1* and *pdi6-2* knockdown mutants display increased resistance to high light, reduced photoinhibition, and an accelerated rate of D1 synthesis (Wittenberg et al., 2014). Therefore, it was proposed that PDI6/PDIL1-2 may function as an attenuator of D1 synthesis during PSII repair (Figure 2).

Thioredoxins are small proteins that contain a redox-active thioredoxin domain with the WCGHC active site (Cain et al., 2009). As enzymes, thioredoxins are active in the reduced form and are able to reduce disulfide bonds in protein substrates (Cain et al., 2009). Thioredoxins are important for regulating thiol/disulfide homeostasis inside chloroplasts (Cain et al., 2009). Three M-type thioredoxins (TRX-M1, M2, and M4) have been found to be involved in PSII biogenesis in chloroplasts (Wang et al., 2013). TRX-M1, M2, and M4 are associated with minor PSII assembly intermediate subcomplexes and they interact with PSII core subunits D1, D2, and CP47. Simultaneous inactivation of the three Arabidopsis *TRX-M* genes causes pale-green leaves, reduced PSII activity, decreased accumulation of PSII complexes, and increased accumulation of reactive oxygen species. PSII core proteins D1 and CP47 were found to be able to form redox-sensitive intermolecular disulfide bonds and concurrent loss of the three M-type thioredoxins interrupts the redox status of these PSII core subunits. According to these results, Wang et al. (2013) proposed that the three TRX-M proteins may assist incorporation of CP47 into PSII core complexes (Figure 1).

In addition to PDIases and thioredoxins, a new type of proteins has been found to regulate thiol/disulfide homeostasis and they mainly act as oxidases by converting free thiols on protein substrates into disulfide bridges. One example is Lumen Thiol Oxidoreductase 1 (LTO1), a thylakoid membrane protein with an integral-membrane vitamin K epoxide reductase domain and a soluble disulfide-bond A oxidoreductase-like domain (Feng et al., 2011; Karamoko et al., 2011; Lu et al., 2013). Each of the two domains contains four conserved cysteine residues (a pair of cysteine residues in the CXXC motif and another pair of separate cysteine residues), which are critical for the disulfide-bond-forming activity of LTO1 (Feng et al., 2011). According to membrane topology analysis, Feng et al. (2011) proposed that the eight conserved cysteine residues are positioned on the lumenal side of thylakoid membranes. This led to the hypothesis that LTO1 is involved in formation of the intramolecular disulfide bond in PsbO (Figures 1, 2), which is located on the lumenal side of thylakoid membranes (Karamoko et al., 2011). Consistent with this hypothesis, LTO1 was found to interact with PsbO1 and PsbO2 and catalyze formation of intramolecular disulfide bonds in recombinant PsbO (Karamoko et al., 2011). In line with these observations, the amounts of PsbO, PsbQ, and PsbQ are substantially reduced in the LTO1-deficient Arabidopsis mutants and the mutants display reduced efficiency of PSII photochemistry, increased accumulation of reactive oxygen species, a smaller plant size, and delayed growth (Karamoko et al., 2011; Lu et al., 2013).

RBD1 (rubredoxin 1) is a small iron-containing protein with a C-terminal transmembrane domain and a rubredoxin domain with two redox-active CXXC motifs (Calderon et al., 2013). Homologs of RBD1 have been found in thylakoid membranes but not plasma membranes of cyanobacteria and in thylakoid membranes of green algae and land plants (Shen et al., 2002; Calderon et al., 2013). The *rbd1* knockout mutants in the cyanobacterium *Synechocystis* sp. PCC 6803, the green alga *Chlamydomonas reinhardtii*, and the higher plant Arabidopsis display a substantial reduction or complete loss of PSII activity and photoautotrophy (Calderon et al., 2013). The amounts of PSII core subunits, such as D1, D2, and CP47, are reduced by 40–90% in these mutants while other components of the photosynthetic apparatus, such as PSI, cytochrome *b*_6_*f* complex, and ATP synthase, are not affected. Based on these data, Calderon et al. (2013) proposed that RBD1 is required for the assembly and/or stability of PSII in oxygenic photosynthetic organisms. Further studies are needed to dissect the precise function of RBD1.

### PPIases

Peptide bonds to proline have *cis* and *trans* conformations (Fischer et al., 1998; Ingelsson et al., 2009). Therefore, folding of proteins such as PSII subunits often involves *cis*-*trans* proline isomerization, which is catalyzed by PPIases (He et al., 2004; Romano et al., 2004; Ingelsson et al., 2009). Three PPIase families have been established according to their immunosuppressant ligand specificity: cyclophilins (CYPs), FK506 (tacrolimus)-binding proteins (FKBPs), and parvulins (Fischer et al., 1998; He et al., 2004; Ingelsson et al., 2009). Two CYPs and one FKBP have been found to be important for the assembly, stability, and/or repair of PSII (Järvi et al., 2015).

CYP20-3/ROC4 (20-kDa cyclophilin 3/rotamase cyclophilin 4) is localized in the chloroplast stroma (Lippuner et al., 1994). The Arabidopsis T-DNA insertion mutant of CYP20-3/ROC4 has a normal phenotype and normal PSII function under ambient light (Cai et al., 2008). Under high light, PSII in the *cyp20-3/roc4* mutant exhibits increased photosensitivity and delayed recovery, which caused growth retardation and leaf yellowing. Under high light, D1 degradation is not affected in the mutant but repair and reassembly of photodamaged PSII is impaired. According to the *cyp20-3/roc4* mutant phenotype, Cai et al. (2008) proposed that CYP20-3/ROC4 is involved in repair and reassembly of PSII under high light (Figure 2). The PPIase activity of CYP20-3/ROC4 makes it a good candidate for catalyzing correct folding of PSII proteins during the repair process of PSII. CYP20-3/ROC4 was also reported to link light and redox signals to cysteine biosynthesis and stress acclimation (Dominguez-Solis et al., 2008; Park et al., 2013; Speiser et al., 2015).

CYP38/TLP40 (cyclophilin of 38 kDa/Thylakoid Lumen Protein of 40 kDa) is predominantly confined in the lumen of non-appressed thylakoids (Fulgosi et al., 1998). Full-length CYP38/TLP40 has a bipartite thylakoid lumen targeting transit peptide, a leucine zipper, a phosphatase-binding module, an acid region for protein-protein interaction, and a C-terminal cyclophilin-type PPIase domain (Fulgosi et al., 1998; Sirpiö et al., 2008). CYP38/TLP40 isolated from spinach leaves demonstrates *in vivo* PPIase activity and co-purification with a thylakoid membrane phosphatase (Fulgosi et al., 1998; Vener et al., 1999). In addition, it was reported that CYP38/TLP40 could be released from thylakoid membranes to the thylakoid lumen upon heat stress and the release is associated with activation of dephosphorylation of PSII subunits in thylakoid membranes (Rokka et al., 2000). Therefore, it was proposed that CYP38/TLP40 acts as a phosphatase inhibitor and regulates dephosphorylation of PSII subunits during PSII repair (Figure 2). Consistent with this hypothesis, T-DNA insertions in the Arabidopsis *CYP38/TLP40* gene cause increased phosphorylation of PSII core subunits and increased photosensitivity of PSII activity (Fu et al., 2007; Sirpiö et al., 2008). In addition, CYP38/TLP40 was found to interact with CP47 through its cyclophilin domain (Vasudevan et al., 2012). Dephosphorylation of PSII subunits during light acclimation is carried out by a type 2C protein phosphatase called PSII core phosphatase (PBCP) (Samol et al., 2012). Further studies are needed to investigate whether CYP38/TLP40 interacts with PBCP and inhibits its phosphatase activity. The *cyp38/tlp40* mutants display retarded growth, pale-green leaves, increased accumulation of PSII monomers, and decreased accumulation of PSII supercomplexes, even under low or ambient light (Fu et al., 2007; Sirpiö et al., 2008). In addition, CYP38/TLP40 was found to co-migrate with PSII core monomers in BN-PAGE (Sirpiö et al., 2008). Therefore, it is likely that CYP38 also functions in conversion of PSII core monomers into higher order PSII complexes (Figures 1, 2).

FKBP20-2 (20-kDa FK506-binding protein 2) is located in the thylakoid lumen (Lima et al., 2006). Recombinant FKBP20-2 demonstrates PPIase activity and the C-terminus of FKBP20-2 has a unique pair of cysteine residues which can be reduced by thioredoxin. T-DNA insertions in the Arabidopsis *FKBP20-2* gene cause smaller plant sizes, reduced chlorophyll contents, stunted growth, reduced PSII activity, increased accumulation of PSII monomers and PSII dimers, and decreased accumulation of PSII supercomplexes under normal light conditions (Lima et al., 2006). Under higher light, the difference in PSII activity between the mutant and the wild type is more pronounced. According to the mutant phenotype, Lima et al. (2006) proposed that FKBP20-2 functions in formation of PSII-LHCII supercomplexes under normal and high light (Figures 1, 2).

### Protein kinases

Multiple studies have shown that high light induces phosphorylation of PSII core proteins, such as D1, D2, CP43, and PsbH (Rintamaki et al., 1997; Vener et al., 2001), which facilitates migration of photodamaged PSII complexes from grana stacks to stroma lamellae (Tikkanen et al., 2008; Goral et al., 2010). Two serine/threonine protein kinases were found to be localized the thylakoid membranes: STN7 (state transition 7) and STN8 (state transition 8) (Bellafiore et al., 2005; Bonardi et al., 2005; Nath et al., 2013). Light-induced phosphorylation of PSII core proteins is carried out by STN8, and to a lesser degree under low light also by STN7 (Figure 2; Bonardi et al., 2005; Tikkanen et al., 2008; Nath et al., 2013). The primary role of STN7 is phosphorylation of LHCII proteins, which leads to displacement of LHCII from PSII to PSI (Bellafiore et al., 2005). Phosphorylation of PSII core proteins promotes unfolding of grana stacks and migration of photodamaged PSII complexes from grana stacks to stroma-exposed thylakoids. This allows easier access of membrane or membrane-associated proteases and co-translational integration of D1 and therefore facilitates repair of photodamaged PSII complexes and proteins (Bonardi et al., 2005; Tikkanen et al., 2008; Khatoon et al., 2009; Goral et al., 2010; Herbstová et al., 2012; Tikkanen and Aro, 2012).

### Protein phosphatases

While the migration of photodamaged PSII complexes is assisted by phosphorylation of PSII core proteins, dephosphorylation of D1 is necessary for efficient turnover of photodamaged D1 in stroma lamellae (Järvi et al., 2015). Two chloroplast protein phosphatases, PBCP and TLP18.3 (Thylakoid Lumen Protein of 18.3 kDa), have demonstrated *in vivo* or *in vitro* phosphatase activity toward PSII core proteins (Sirpiö et al., 2007; Wu et al., 2011; Samol et al., 2012). PBCP is a type 2C protein phosphatase predominantly found in the chloroplast stroma, with a minor fraction associated with thylakoid membranes (Samol et al., 2012). Compared to wild-type Arabidopsis, the PBCP-deficient mutants display delayed dephosphorylation of PSII core proteins (D1, D2, CP43, and PsbH) and normal dephosphorylation of LHCII proteins, upon exposure to far-red light, which favors PSI excitation and dephosphorylation of thylakoid proteins (Samol et al., 2012). Samol et al. (2012) concluded that PBCP is required for efficient dephosphorylation of PSII core proteins (Figure 2).

TLP18.3 is a thylakoid membrane protein with the N-terminal domain of unknown function located in the thylakoid lumen (Sirpiö et al., 2007). It was originally identified as an auxiliary protein involved in dimerization of PSII monomers and degradation of photodamaged D1 (Figures 1, 2). The TLP18.3-deficient Arabidopsis mutants do not show a clear visual phenotype under normal growth conditions but exhibit retarded growth under fluctuating light (Sirpiö et al., 2007), suggesting that TLP18.3 is more important to PSII repair than to *de novo* PSII assembly. Compared to the wild type, the TLP18.3-deficient mutants have fewer PSII dimers and more PSI monomers under normal and fluctuating light. In addition, the rate of high-light-induced D1 turnover is ~50% slower in the TLP18.3-deficient mutants. Consistent with its dual roles in dimerization of PSII monomers, which occurs in grana stacks, and degradation of photodamaged D1, which takes place in stroma lamellae, TLP18.3 was found evenly distributed between grana stacks and stroma lamellae (Sirpiö et al., 2007). It was later found that the domain of unknown function in TLP18.3 possesses acid phosphatase activity toward synthetic phosphorylated oligopeptides that resemble the phosphorylation sites of PSII core proteins D1 and D2 (Wu et al., 2011). However, how the acid phosphatase activity of TLP18.3 is related to the role of TLP18.3 in PSII assembly and repair is still not clear (Figure 2): the phosphorylation sites of D1 are exposed to the stroma side while the acid phosphatase domain of TLP18.3 is located in the thylakoid lumen.

Dephosphorylation of LHCII proteins, such as LHCB1 and LHCB2, is carried out by PPH1/TAP38 (Protein Phosphatase 1/Thylakoid-Associated Phosphatase of 38 kDa) (Pribil et al., 2010; Shapiguzov et al., 2010). PPH1/TAP38 is a type 2C protein phosphatase with a C-terminal single-pass transmembrane domain; it is predominantly located in stroma lamellae and grana margins, where active dephosphorylation of LHCII and PSII core proteins occurs. Loss-of-function mutations in the Arabidopsis *PPH1/TAP38* gene causes decreased dephosphorylation of LHCII while overexpression of PPH1/TAP38 enhances dephosphorylation of LHCII (Pribil et al., 2010; Shapiguzov et al., 2010). The phosphorylation status of PSII core proteins is largely unaffected in the *pph1/tap38* mutants, suggesting that the primary function of PPH1/TAP38 is dephosphorylation of LHCII proteins. Recombinant PPH1/TAP38 is able to dephosphorylate LHCII directly, in an *in vitro* assay (Pribil et al., 2010). Reversible phosphorylation of LHCII is important for the movement of LHCII between PSII and PSI, according to the changes in the spectral composition of incident light. Therefore, the catalytic activities of LHCII kinase STN7 and phosphatase PPH1/TAP38 are important for balancing the light absorption capacity between PSI and PSII (Pesaresi et al., 2011).

### FtsH proteases

FtsH proteases are ubiquitous ATP-dependent, zinc metalloendopeptidases (Yu et al., 2004). FtsHs typically consist of an N-terminal double-pass transmembrane domain, an ATPase domain, and a C-terminal zinc-binding site. Crystal structures of the ATPase domain of bacterial FtsHs and single-particle electron cryo-microscopy analysis of cyanobacterial FtsHs showed that FtsHs exist as ringlike hexamers (Krzywda et al., 2002; Niwa et al., 2002; Boehm et al., 2012b). Bacteria contain one *FtsH* gene and the FtsH protein forms homohexamers while cyanobacteria and eukaryotes have multiple *FtsH* genes and the FtsH proteins form heterohexamers (Mann et al., 2000; Krzywda et al., 2002; Niwa et al., 2002; Zaltsman et al., 2005b; Boehm et al., 2012b). The Arabidopsis genome encodes 12 FtsH proteases; eight FtsHs (FtsH1, FtsH2/VAR2, FtsH5/VAR1—FtsH9, and FtsH12; VAR1 stands for Yellow Variegated 1) were verified experimentally to be chloroplast-targeted; FtsH11 was showed to be dual targeted to the chloroplast (possible thylakoid membranes) and the inner mitochondria membrane (Chen et al., 2000; Takechi et al., 2000; Sakamoto et al., 2002, 2003; Urantowka et al., 2005). FtsH2/VAR2 and FtsH5/VAR1 were found to be localized to thylakoid membranes, with their catalytic domain facing the stromal side of the membrane (Chen et al., 2000; Sakamoto et al., 2003). Among the nine chloroplast- or dual-targeted FtsHs, the functions of FtsH1, FtsH2/VAR2, FtsH5/VAR1, FtsH6, FtsH8, and FtsH11 have been explored experimentally. FtsH and Deg proteases have been known to be involved in degradation of photodamaged D1. Early *in vitro* studies suggested that this is a two-step process including the initial cleavage at the stromal DE loop via Deg2 and the subsequent removal of the N-terminal fragment by FtsHs (Lindahl et al., 1996, 2000; Spetea et al., 1999; Haubühl et al., 2001). It was later proposed that FtsHs play a more important role than Deg proteases in D1 turnover (Silva et al., 2003; Nixon et al., 2005, 2010; Huesgen et al., 2009; Kato et al., 2012; Komenda et al., 2012a).

Loss-of-function mutations in the Arabidopsis *FtsH2/VAR2* or *FtsH5/VAR1* gene cause variegated leaves (Sakamoto et al., 2002, 2003; Yu et al., 2004, 2005; Zaltsman et al., 2005a,b; Kato et al., 2007, 2009, 2012; Wagner et al., 2011). The green sectors in the *ftsh2/var2* or *ftsh5/var1* mutants are formed by cells with normal chloroplasts and the white leaf sectors are formed by viable cells with undifferentiated plastids (Chen et al., 2000; Sakamoto et al., 2002; Kapri-Pardes et al., 2007). These data suggest that FtsH2/VAR2 and FtsH5/VAR1 are required for chloroplast biogenesis and thylakoid formation (Chen et al., 2000; Sakamoto et al., 2002; Zaltsman et al., 2005a,b; Kapri-Pardes et al., 2007).

Compared to wild-type leaves, the green leaf sectors of the *ftsh2/var2* or *ftsh5/var1* mutants demonstrate increased photosensitivity and delayed recovery of PSII activity (Zaltsman et al., 2005a,b). Chloroplasts in the green sectors of the *ftsh2/var2* or *ftsh5/var1* mutants accumulate fewer PSII supercomplexes, more PSII subcomplexes, and more reactive oxygen species than wild-type chloroplasts (Kato et al., 2009). These observations are due to the proteolytic activity of FtsHs toward photodamaged D1 (Bailey et al., 2002; Kato et al., 2009). Because the variegated phenotype complicates biochemical analyses, Kato et al. (2009) used another mutation, *fu-gaeri1* (*fug1*), to suppress leaf variegation, and generated the non-variegated *ftsh2/var2 fug1* and *ftsh5/var1 fug1* plants. Compared to the *fug1* single mutant, photodamaged D1 is not replaced in the *ftsh2/var2 fug1* and *ftsh5/var1 fug1* mutants, under different light intensities. Taken together, these data show that FtsH2/VAR2 and FtsH5/VAR1 play an important role at the early stage of D1 turnover and not just in the subsequent removal of D1 degradation products (Figure 2; Bailey et al., 2002; Kato et al., 2009).

The phenotypes observed in the *ftsh2/var2* or *ftsh5/var1* mutants are absent in the *ftsh1, ftsh6, ftsh8*, and *ftsh11* single mutants under normal or high-light conditions (Sakamoto et al., 2003; Zaltsman et al., 2005b; Chen et al., 2006b; Wagner et al., 2011). These findings suggest that FtsH2/VAR2 and FtsH5/VAR1 play a dominant role in chloroplast biogenesis, thylakoid formation, and PSII repair (Sakamoto et al., 2003). Phylogenetic analysis showed that *FtsH1* and *FtsH5* are two duplicated genes (subunit type A), so are *FtsH2* and *FtsH8* (subunit type B) (Yu et al., 2004, 2005; Zaltsman et al., 2005b). The phenotype of the *ftsh2/var2* mutant can be restored by overexpression of *FtsH8*; the *ftsh2/var2 ftsh8* double mutant is infertile; and the *ftsh1 ftsh5/var1* double mutant resembles the *ftsh2/var2 ftsh8* double mutant (Yu et al., 2004; Zaltsman et al., 2005b). These data suggest that FtsH1 and FtsH5/VAR1 are interchangeable, so are FtsH2/VAR2 and FtsH8, and that the presence of two types of FtsH subunits is necessary for chloroplast biogenesis, thylakoid formation, and PSII repair (Figure 2; Zaltsman et al., 2005b).

FtsH6 was reported to participate in degradation of LHCII in Arabidopsis leaves during high-light acclimation and senescence (Zelisko et al., 2005). Using an *in vitro* degradation system (i.e., isolated thylakoid membranes), Zelisko et al. (2005) showed that, compared to the wild type, the *ftsh6* knockout mutant has reduced degradation of LHCB1 after high-light acclimation and reduced degradation of LHCB3 after dark-induced senescence. However, *in vivo* degradation of LHCII proteins does not appear to be impaired in the *ftsh6* knockout mutants (Wagner et al., 2011). Under various conditions, including high-light acclimation and dark-induced senescence, the abundances of LHCB1 and LHCB3 in the *ftsh6* knockout mutants are not statistically different from those in the wild type. Further investigation is needed to understand the precise role of FtsH6.

FtsH11 was reported to be critical in thermoprotection of the photosynthetic apparatus (Chen et al., 2006b; Wagner et al., 2011). When exposed to temperatures above 30°C, which are permissive for wild-type Arabidopsis, the growth and development of the *ftsh11* mutants is arrested (Chen et al., 2006b). Compared to the wild type under the same high-temperature treatment, the *ftsh11* mutants have reduced levels of chlorophyll and reduced PSII activity. Consistent with the hypothesis that FtsH11 is involved in thermotolerance, the expression of the *FtsH11* gene is up-regulated by high temperature (Chen et al., 2006b).

### Deg proteases

Deg proteases are ubiquitous ATP-independent, serine endopeptidases (Schuhmann and Adamska, 2012). The Arabidopsis genome encodes 16 Deg proteases, five of which are peripherally attached to thylakoid membranes: two (Deg2 and Deg7) on the stroma side and three (Deg1, Deg5, and Deg8) on the lumenal side (Huesgen et al., 2005; Schuhmann and Adamska, 2012). These five chloroplast-localized Deg proteases have been proposed to be involved in degradation of photodamaged D1 (Schuhmann and Adamska, 2012). In addition to the trypsin-like protease domain, most Deg proteases, such as Deg1, Deg2, Deg7, and Deg8, have at least one PDZ domain for protein-protein interactions. It is conceivable that these chloroplast-localized PDZ domain-containing Deg proteases may act as chaperones and function in assembly of the photosynthetic apparatus (Sun et al., 2010b; Schuhmann and Adamska, 2012).

Arabidopsis RNA interference (RNAi) lines of Deg1 have a smaller plant size, increased sensitivity of PSII activity to high light, and increased accumulation of non-degraded (and presumably photodamaged) D1 (Kapri-Pardes et al., 2007). The RNAi lines display decreased accumulation of the 16- and 5.2-kDa C-terminal degradation products of D1, which correspond to the cleavage products at the lumenal CD loop, and immediately after the transmembrane helix E, respectively. The addition of recombinant Deg1 into inside-out thylakoid membranes isolated from the Deg1-deficient plants induces formation of the 5.2-kDa C-terminal degradation product of D1. These data suggest that Deg1 is involved in degradation of photodamaged D1 (specifically, the cleavage at the lumenal CD loop immediately downstream of the transmembrane helix E) in PSII repair (Figure 2; Kapri-Pardes et al., 2007). Unfortunately, D1 turnover was not assessed in Deg1 RNAi lines. Deg1 is capable of degrading lumenal proteins plastocyanin and PsbO, suggesting that Deg1 may also acts as a general purpose endopeptidase in the thylakoid lumen (Chassin et al., 2002).

Because Deg1 has a PDZ domain for protein-protein interactions, Sun et al. (2010b) investigated whether Deg1 functions as a chaperone during assembly of PSII complexes (Sun et al., 2010b). Deg1 was found to co-migrate with D1 in BN-PAGE and pull down D1, D2, CP43, and CP47 in an immunoprecipitation assay. Recombinant Deg1 has the ability to fold reduced and denatured protein substrates in the presence of both reduced and oxidized glutathione. The RNAi lines of Deg1 display reduced accumulation of PSII complexes and normal accumulation of other photosynthetic complexes. In addition, assembly of newly synthesized PSII subunits into PSII dimers and PSII supercomplexes is hindered in the RNAi lines, although synthesis of the corresponding proteins in chloroplasts is not impaired. Based on these results, Sun et al. (2010b) proposed that Deg1 also acts as a chaperone and functions in the integration of newly synthesized PSII subunits, such as D1, D2, CP43, and CP47, into PSII complexes (Figures 1, 2). However, these experimental data do not rule out the possibility that Deg1 is directly involved in degradation of these PSII subunits.

Deg2 has a PDZ domain and a short hydrophobic segment, in addition to the trypsin-like protease domain (Haubühl et al., 2001). Recombinant Deg2 demonstrates proteolytic activity toward photodamaged D1 and is able to produce the 23-kDa N-terminal and the 10-kDa C-terminal degradation products, which correspond to the cleavage products at the stromal DE loop (Haubühl et al., 2001). Although recombinant Deg2 is proteolytically active, the *deg2* knockout mutants have the same plant morphology, PSII activity, and D1 turnover rate as wild-type Arabidopsis, under normal or elevated light (Huesgen et al., 2006). Therefore, it was proposed that Deg2 functions as a minor protease in *in vivo* degradation of photodamaged D1 (Figure 2). Deg2 was also reported to be involved in stress-induced degradation of LHCB6 (Luciński et al., 2011b).

Deg5 and Deg8 form heterohexamers and the Deg5-to-Deg8 ratio is ~1:1 (Sun et al., 2007). Deg5 is ~120 amino acids shorter than Deg1 and Deg8 and it does not contain any PDZ domain (Schuhmann and Adamska, 2012). Although Deg8 has a PDZ domain, it shows no chaperone activity (i.e., PDIase activity) toward reduced and denatured protein substrates (Sun et al., 2010b). Recombinant Deg8 demonstrates proteolytic activity toward photodamaged D1 and is able to produce the 16-kDa N-terminal and the 18-kDa C-terminal degradation products, which correspond to the cleavage products at the lumenal CD loop (Sun et al., 2007). Although only recombinant Deg8 is proteolytically active, the *deg5* and *deg8* single knockout mutants of Arabidopsis both display impaired degradation of newly synthesized D1 and the impairment is more pronounced in the *deg5 deg8* double mutant (Sun et al., 2007). The defect in D1 turnover in the mutants is reflected in PSII activity. PSII in the *deg5* and *deg8* single mutants exhibits increased sensitivity to high light and the sensitivity is more obvious in the *deg5 deg8* double mutant. Under normal light, the two single mutants and the double mutant have a normal phenotype. Based on these data, Sun et al. (2007) proposed that Deg5 and Deg8 act as heterohexameric endopeptidases and cleave photodamaged D1 at the lumenal CD loop during PSII repair (Figure 2). Deg5 has also been reported to be involved in wounding-related disposal of PsbF (Luciński et al., 2011a).

Deg7 (1097 amino acids at full length) is twice as long as most Deg proteases; it has two trypsin-like protease domains (one active and one degenerated) and four PDZ domains (three active and one degenerated) (Schuhmann et al., 2011). The domain composition suggests that Deg7 is the result of a whole-gene duplication event followed by subsequent degeneration (Schuhmann et al., 2011). Deg7 forms homotrimers and the oligomerization is mediated through the degenerated protease domain (Schuhmann et al., 2011). Recombinant Deg7 demonstrates proteolytic activity toward photodamaged D1, D2, CP43, and CP47 (Sun et al., 2010a). Compared to wild-type Arabidopsis, the *deg7* null mutant displays retarded growth, reduced PSII activity, and reduced degradation of PSII core proteins D1, D2, CP43, and CP47 under high light. However, under normal light, there is no apparent difference in plant growth or morphology between the *deg7* mutant and the wild type. These data suggest that Deg7 is involved in cleavage of photodamaged PSII core proteins D1, D2, CP47, and CP43 from the stroma side during PSII repair (Figure 2; Sun et al., 2010a).

### HCF243, PSB27-H1, and PSB27-H2/LPA19 in biogenesis, C-terminal processing, and/or assembly of D1

HCF243 (High Chlorophyll Fluorescence 243) is an intrinsic thylakoid membrane protein with no recognizable domain or motif (Zhang et al., 2011). A loss-of-function mutation in the Arabidopsis *HCF243* gene causes substantial reductions in accumulation of PSII core subunits (D1, D2, CP43, and CP47) and assembly of PSII complexes (Zhang et al., 2011). Unlike PSII core subunits, the amounts of extrinsic PSII subunits, PSII antenna proteins, and other non-PSII thylakoid membrane proteins are hardly affected in the *hcf243* mutant. In line with these observations, PSII activity is dramatically reduced in the *hcf243* mutant and the mutant has pale-green leaves and a much smaller plant size than the wild type. Pulse-labeling experiments indicated that these defects are caused by the severely reduced synthesis of D1 and to a lesser degree of D2 (Zhang et al., 2011). Indeed, the *hcf243* mutant over-accumulates pD1, the D1 precursor with an unprocessed C-terminus. HCF243 was also found to interact with D1 *in vivo* (Zhang et al., 2011). These data suggest that HCF243 is involved in biogenesis, processing, and assembly of D1 and possible biogenesis of D2 as well (Figures 1, 2).

PSB27s (PSII protein 27s) are thylakoid lumen proteins peripherally attached to thylakoid membranes (Chen et al., 2006a; Wei et al., 2010). Cyanobacterial Psb27 was found to facilitate assembly of the OEC manganese and plays a role in PSII repair (Nowaczyk et al., 2006; Liu et al., 2011a,b; Komenda et al., 2012b). The Arabidopsis genome encodes two PSB27s: PSB27-H1 and PSB27-H2/LPA19 (LPA19 stands for Low PSII Accumulation 19). Under normal light, the PSII protein composition is not changed and PSII activity is only slightly reduced in the Arabidopsis T-DNA insertion mutant of PSB27-H1 (Chen et al., 2006a; Dietzel et al., 2011). However, the *psb27-H1* mutant has reduced amounts of PSII-LHCII supercomplexes, suggesting that PSB27-H1 is required for the formation and stability of PSII-LHCII supercomplexes (Figure 1; Dietzel et al., 2011). Under high light, PSII activity and the amount of D1 decrease much faster in the *psb27-H1* mutant than in the wild type. In addition, the *psb27-H1* mutant displays delayed recovery of PSII activity after photoinhibition, suggesting that PSB27-H1 is involved in the repair cycle of photodamaged PSII. Unlike *psb27-H1*, the *psb27-H2/lpa19* mutants of Arabidopsis have pale-green leaves, a smaller plant size, and reduced PSII activity even under normal light conditions (Wei et al., 2010). Pulse-labeling experiments showed that C-terminal processing of D1 is impaired in the *psb27-H2/lpa19* null mutants. PSB27-H2/LPA19 was found to specifically interact with the soluble C-terminus of precursor and mature D1. It was concluded that PSB27-H2/LPA19 functions in C-terminal processing of D1 (Figure 1; Wei et al., 2010).

### HCF136, PAM68, and PSBN/PBF1 in assembly OF PSII reaction complexes

HCF136 (High Chlorophyll Fluorescence 136) is a lumenal protein found in stroma lamellae; it contains no recognizable domain or motif (Meurer et al., 1998). The cyanobacterial homolog of HCF136, YCF48 (hypothetical chloroplast reading frame number 48), was found to interact with pD1, and to a lesser degree, partially processed and unassembled D1, but not with mature and unassembled D1 or D2, in a split-ubiquitin yeast-two-hybrid assay (Komenda et al., 2008). In addition, higher-plant HCF136 was found to be associated with the PSII precomplex D2-Cyt *b*_559_ and PSII reaction-center complexes RC, RC47a, and RC47b (Plücken et al., 2002). The HCF136-deficient Arabidopsis mutant is able to accumulate PSI and cytochrome *b*_6_*f* complex proteins, but unable to accumulate PSII proteins, perform PSII activity, or grow photoautotrophically (Meurer et al., 1998; Plücken et al., 2002). Pulse-labeling studies showed that the *hcf136* mutant is defective in biogenesis of PSII minimal reaction-center complexes, not in biosynthesis of PSII proteins (Meurer et al., 1998; Plücken et al., 2002). Therefore, it was proposed that HCF136 is involved in assembly of PSII reaction-center complexes such as RC, RC47a, and RC47b (Figure 1). Consistent with this hypothesis, HCF136 was found to interact with another PSII assembly factor, PAM68 (Armbruster et al., 2010).

PAM68 in vascular plants is an integral thylakoid membrane protein with an acidic domain and a double-pass transmembrane domain (Armbruster et al., 2010). PAM68 was found to be associated with LMM complexes that are formed at an early step of PSII assembly (Armbruster et al., 2010). These complexes contain D1, D2, and LPA1/PratA, which may correspond to PSII minimal reaction-center complexes. PAM68 was found to interact with a number of PSII core subunits (D1, D2, CP43, CP47, PsbH, and PsbI) and PSII assembly factors (ALB3, HCF136, and LPA1/PratA, and LPA2). The PAM68-deficient Arabidopsis mutants have pale-green leaves, drastically reduced PSII activity, and severely retarded growth, under normal growth conditions. Consistent with these observations, the *pam68* mutants have severely reduced amounts of PSII core subunits, and they over-accumulate PSII reaction-center complexes at the expense of higher order PSII complexes. PAM68 was therefore proposed to be necessary for converting PSII minimal reaction-center complexes into larger PSII complexes (Figure 1; Armbruster et al., 2010). The *pam68* mutants were also found to over-accumulate pD1 (Armbruster et al., 2010), suggesting that PAM68 is also necessary for efficient C-terminal processing of D1 (Figures 1, 2).

PsbN is a small thylakoid membrane protein encoded by the plastid genome (Krech et al., 2013; Torabi et al., 2014). It was originally thought as a PSII subunit (Ikeuchi et al., 1989); however, two recent studies showed that PsbN is not a PSII subunit, but an assembly factor of PSII (Krech et al., 2013; Torabi et al., 2014). Thus, Krech et al. (2013) proposed a new name, Photosystem Biogenesis Factor 1 (PBF1) for this protein. Tobacco (*Nicotiana tabacum*) homoplastomic Δ*psbN/pbf1* mutants have pale-green leaves and slow autotrophic growth (Krech et al., 2013). Pulse-labeling and two-dimensional gel electrophoresis showed that formation of PSII precomplexes, e.g., pD1-PsbI and D2-Cyt *b*_559_, is not affected in the Δ*psbN/pbf1* mutants but assembly of PSII minimal reaction-center complexes and higher order PSII complexes is hampered (Torabi et al., 2014). It was concluded that PsbN/PBF1 is involved in formation of PSII minimal reaction-center complexes (Figure 1). The Δ*psbN/pbf1* mutants are extremely sensitive to light, even at relatively low light and PSII in the Δ*psbN/pbf1* mutants is unable to recover from photoinhibition (Torabi et al., 2014). These data indicate that PsbN/PBF1 may also function in PSII repair, which occurs in stroma lamellae. Consistent with this hypothesis, PsbN was found to be predominantly located in stroma lamellae (Torabi et al., 2014). Loss of PsbN changes the phosphorylation status of PSII core proteins and LHCII proteins, which is important for the migration of photodamaged PSII complexes from grana stacks to stroma-exposed thylakoids, and for balancing the light absorption capacity between PSI and PSII, respectively. Therefore, it is possible that PsbN is involved in PSII repair by regulating the phosphorylation status of PSII core proteins and LHCII proteins (Figure 2).

### PSB28 in biogenesis of chlorophyll-binding proteins such as CP47, PsaA, and PsaB

PSB28 (PSII protein 28) is a small protein predominantly localized in the stroma/cytoplasm (Shi et al., 2012). In *Synechocystis* sp. PCC 6803, a small fraction of Psb28 was found to be associated with unassembled CP47, RC47, and PSII monomers at the stroma side (Dobáková et al., 2009; Boehm et al., 2012a). Deletion of this protein in *Synechocystis* sp. PCC 6803 cause a decreased amount of unassembled CP47 and increased amounts of PSII minimal reaction-center complexes and unassembled D1 (Dobáková et al., 2009). In addition, the Δ*psb28* mutant exhibits reduced synthesis of CP47 and PsaA/PsaB heterodimers. Therefore, Psb28 was considered to function in biogenesis and assembly of chlorophyll-containing proteins such as CP47, PsaA, and PsaB in cyanobacteria (Dobáková et al., 2009). Little is known about the role of PSB28 in photosynthetic eukaryotes except that PSB28 does exist in higher plants and that the absence of PSB28 results in a pale-green phenotype in rice (Jung et al., 2008; Mabbitt et al., 2014). However, because PSB28 is evolutionary conserved, it is reasonable to predict that PSB28 in higher plants may also function in biogenesis and assembly of chlorophyll-containing proteins such as CP47 (Figure 1).

### LPA2 and LPA3 in synthesis and assembly of CP43

LPA2 is a small intrinsic thylakoid membrane protein with a C-terminal double-pass transmembrane domain (Ma et al., 2007). LPA3 (Low PSII Accumulation 3) is a chloroplast protein without any transmembrane domain or any other recognizable domain or motif. However, sub-chloroplast fractionation revealed that LPA3 could be located in chloroplast stroma or associated with thylakoid membranes (Cai et al., 2010). Although LPA2 and LPA3 are not homologous, they were identified as two auxiliary proteins assisting incorporation of CP43 into PSII via interaction with cpSRP translocase ALB3 (Ma et al., 2007; Cai et al., 2010). LPA2 and LPA3 were found to interact with CP43, ALB3, and each other (Cai et al., 2010). Pulse-labeling experiments showed that assembly from CP43-less reaction-center complexes to PSII monomers and formation of PSII-LHCII supercomplexes are distinctively slower in the *lpa2* and *lpa3* single mutants than in wild-type Arabidopsis (Ma et al., 2007; Cai et al., 2010). Synthesis of CP43 is greatly reduced in the *lpa2* and *lpa3* single mutants while synthesis of other PSII core subunits D1, D2, and CP47 is comparable between the single mutants and the wild type (Ma et al., 2007; Cai et al., 2010). LPA2 and LPA3 appear to be functional redundant because the *lpa2 lpa3* double mutant has no detectable amounts of D1, D2, CP43, and CP47 and is seedling-lethal. Taken together, LPA2 and LPA3 were proposed to be involved in synthesis and assembly of CP43 (Figures 1, 2; Ma et al., 2007; Cai et al., 2010).

### PSB33 in association of LHCII with PSII

PSB33 (PSII protein 33) is a thylakoid membrane protein with an N-terminal Rieske-type domain exposed to the stroma side, a double-pass transmembrane domain, and a C-terminal partial chlorophyll-binding domain (Fristedt et al., 2015). PSB33 was found to co-migrate with PSII-LHCII supercomplexes, PSII dimers, PSII monomers, and CP43-less PSII monomers in BN-PAGE (Fristedt et al., 2015). Loss-of-function mutations in the Arabidopsis *PSB33* gene cause reduced amounts of PSII-LHCII supercomplexes, and increased amounts of PSII dimers, lower state transition, lower non-photochemical quenching, increased photosensitivity, and retarded growth (Fristedt et al., 2015). According to these data, Fristedt et al. (2015) proposed that PSB33 may mediate the association of LHCII with PSII core complexes (Figure 1) and balance the light absorption capacity between PSII and PSI. It is likely that PSB33 also functions in attaching LHCII to PSII during reassembly of repaired PSII (Figure 2), because the defect in PSII activity in the *psb33* mutants is more pronounced under higher light (Fristedt et al., 2015).

### HHL1 and MPH1 in protection of PSII from photodamage

HHL1 (Hypersensitive to High Light 1) is a thylakoid membrane protein with a single-pass transmembrane domain, and a C-terminal partial von Willebrand factor type A domain, which is known to mediate protein-protein interactions (Jin et al., 2014). Under high light, the HHL1-deficient mutants have lower efficiency of PSII photochemistry, lower amounts of PSII core subunits and PSII-LHCII supercomplexes, and higher amounts of reactive oxygen species, than wild-type Arabidopsis (Jin et al., 2014). Many of these defects become milder under normal light. Therefore, it was proposed that HHL1 is involved in the repair and reassembly cycle of photodamaged PSII. Consistent with this hypothesis, HHL1 was found in both grana stacks and stroma lamellae, and PSII core subunits in thylakoid membranes isolated from HHL1-deficient plants were found to be less stable than those isolated from wild-type plants (Jin et al., 2014). Jin et al. (2014) also observed *in vivo* and *in vitro* interaction between HHL1 and LQY1, another protein involved in PSII repair. Because the majority of HHL1 is associated with PSII core monomers, it is likely that HHL1 “collaborates” with LQY1 and assists the reassembly of PSII core monomers and PSII-LHCII supercomplexes during PSII repair (Figure 2). In line with this hypothesis, the *hhl1 lqy1* double mutant is more sensitive to high light than the single mutants (Jin et al., 2014).

MPH1 (Maintenance of PSII under High light 1) is a proline-rich intrinsic thylakoid membrane protein with a single-pass transmembrane domain; it is present in grana stacks, grana margins, and stroma lamellae (Liu and Last, 2015a,b). Under normal light, PSII activity and the composition of PSII complexes in MPH1-deficient mutants are similar to those in wild-type Arabidopsis. Under high light, the *mph1* mutants have lower efficiency of PSII photochemistry, and lower amounts of PSII-LHCII supercomplexes, PSII dimers, and PSII core monomers, than the wild type. Therefore, it was proposed that MPH1 has a role in protection and/or stabilization of PSII under high light (Liu and Last, 2015a,b). Consistent with this hypothesis, MPH1 was found to change its association under different light intensities: under normal light, the majority of MPH1 is associated with PSII core monomers; under high light, the majority of MPH1 becomes associated with PSII-LHCII supercomplexes (Liu and Last, 2015a). Because of the interactions between MPH1 and different PSII complexes under different light irradiance and the reductions in the amounts of PSII monomers and higher order PSII complexes, it is likely that MPH1 is involved in the assembly and/or stability of PSII core monomers and higher order PSII complexes under high light (Figure 2).

## Concluding remarks

Photosynthesis directly or indirectly provides chemical energy for nearly all life forms on earth. Due to the importance of photosynthesis, the structure, biogenesis, and maintenance of the photosynthetic apparatus have long been one of the major focuses of research. The combination of proteomics, X-ray crystallography, and single-particle electron cryo-microscopy approaches has led to a comprehensive understanding of the structure and subunit composition of PSII. In addition, significant progresses have been made in the identification and functional studies of protein factors that are involved in *de novo* assembly and/or the repair and reassembly cycle of PSII. The inclusion of thylakoid protein trafficking/targeting systems and enzymes that catalyze important enzymatic steps, along with various assembly/stability factors allows a more comprehensive view of recent advances in this field. However, additional efforts are in great need to (1) dissect the precise functions of understudied assembly/stability factors or enzymes; and (2) build a protein interactome network that would provide a systems view of the interplay among different assembly/stability factors, enzymes, thylakoid protein trafficking/targeting systems, PSII assembly and repair complexes, and PSII subunits.

## Author contributions

This review was written entirely by YL.

### Conflict of interest statement

The author declares that the research was conducted in the absence of any commercial or financial relationships that could be construed as a potential conflict of interest.

## Abbreviations

ALB3: Albino3
BN-PAGE: blue native-polyacrylamide gel electrophoresis
CP43: Photosystem II chlorophyll protein of 43 kDa, also known as PsbC
CP47: Photosystem II chlorophyll protein of 47 kDa, also known as PsbB
CPRabA5e: chloroplast Rab GTPase A5e
cpFtsY: filamentation temperature sensitive protein Y
cpSec: chloroplast secretory
cpSecA, cpSecE, cpSecY: chloroplast secretory translocase A, E, and Y
cpSRP: chloroplast signal recognition particle
cpSRP43 and cpSRP54: chloroplast Signal Recognition Particle protein of 43 and 54 kDa
cpTat: chloroplast twin-arginine translocation
cpTatC: chloroplast twin-arginine translocation protein C
CtpA1 and CtpA2: C-terminal processing peptidases A 1 and 2
CYO1: Shiyou 1 (Shiyou means cotyledon in Japanese), also known as SCO2
CYP20-3: 20-kDa cyclophilin 3
CYP38: cyclophilin of 38 kDa
Cyt *b*_559_: cytochrome *b*_559_
Deg1, Deg2, Deg5, Deg7, and Deg8: Degradation-of-periplasmic-proteins proteases 1, 2, 5, 7, and 8
FKBP20-2: 20-kDa FK506 (tacrolimus)-binding protein
ELIP1 and ELIP2: early light-induced proteins 1 and 2
FtsH1, FtsH2, FtsH5, FtsH6, FtsH8, FtsH9, FtsH11, and FtsH12: filamentation temperature sensitive proteins H 1, 2, 5, 6, 8, 9, 11, and 12; FtsH2 and FtsH5 are also known as VAR2 and VAR1, respectively
fug1: fu-gaeri1
GTPase: GTP hydrolase
HCF106, HCF136, HCF173, HCF243, and HCF244: High Chlorophyll Fluorescence 106, 136, 173, 243, and 244
HHL1: Hypersensitive to High Light 1
HLIP: high-light-induced protein
LHCII: light-harvesting complex II
LHCB1, LHCB2, LHCB3, LHCB4, LHCB5, and LHCB6: Photosystem II light-harvesting chlorophyll *a*/*b*-binding proteins 1, 2, 3, 4, 5, and 6
LHCP: light-harvesting chlorophyll *a*/*b*-binding protein
LIL3:1 and LIL3:2: light-harvesting-like proteins 3:1 and 3:2
LMM: low molecular mass
LPA1, LPA2, LPA3, and LPA19: Low Photosystem II Accumulation 1, 2, 3, and 19; LPA1 is also known as PratA
LQY1: Low Quantum Yield of Photosystem II 1
LTO1: Lumen Thiol Oxidoreductase 1
MET1: Mesophyll-Enriched Thylakoid protein 1
MPH1: Maintenance of Photosystem II under High light 1
OEC: oxygen-evolving complex
OHP1 and OHP2: one-helix proteins 1 and 2
PAM68: Photosynthesis Affected Mutant 68
PBCP: Photosystem II core phosphatase
PBF1: Photosystem Biogenesis Factor 1
pD1: precursor D1
PDI6: Protein Disulfide Isomerase 6, also known as PDIL1-2
PDIase: protein disulfide isomerase
PDIL1-2: Protein Disulfide Isomerase-Like 1-2, also known as PDI6
PPH1: Protein Phosphatase 1
PPIase: Peptidylprolyl Isomerase
PPL1 and PPL2: PsbP-Like proteins 1 and 2
PratA: Processing-associated tetratricopeptide repeat protein A, also known as LPA1
PSI: Photosystem I
PSII: Photosystem II
Psa: Photosystem II
PsaH and PsaK: Photosystem I proteins H and K
Psb: Photosystem II
PSB28, PSB29, and PSB33: Photosystem II proteins 28, 29 and 33; PSB29 is also known as THF1
PsbA, PsbB, PsbC, PsbD, PsbE, PsbF, PsbH, PsbI, PsbJ, PsbK, PsbL, PsbM, PsbN, PsbO, PsbP, PsbQ, PsbR, PsbT, PsbU, PsbV, PsbW, PsbX, PsbY, PsbZ: Photosystem II protein A, B, C, D, E, F, H, I, J, K, L, M, N, O, P, Q, R, T, U, V, W, X, Y and Z; PsbA, PsbB, PsbC, and PsbD are also known as D1, CP47, CP43, and D2; PsbE and PsbF are also known as cytochrome *b*_559_ subunit alpha and beta, respectively; PsbN is also known as PBF1; PsbO, PsbP, and PsbQ are also known as the 33-, 23-, and 17-kDa protein of the oxygen-evolving complex, respectively
PsbTc: chloroplast-encoded Photosystem II protein T
PsbTn: nuclear-encoded PSII protein T
RC: minimal reaction-center complex which lacks CP47 and CP43
RC47a and RC 47b: reaction-center complexes that contain CP47 but lack CP43
RBD1: rubredoxin 1
RNAi: RNA interference
SCO2: Snowy Cotyledon 2, also known as CYO1
SCP: small chlorophyll-binding-like protein
SDR: short-chain dehydrogenase/reductase
SRP: signal recognition particle
STN7 and STN8: state transition 7 and 8
ROC4: rotamase cyclophilin 4
SEP3.1 and SEP3.2: stress-enhanced protein 3.1 and 3.2
TerC: Tellurite-resistance protein C
T-DNA: transfer DNA
TAP38: Thylakoid-Associated Phosphatase of 38 kDa
THA4: Thylakoid Assembly 4
THF1: Thylakoid Formation 1, also known as PSB29
TLP18.3 and TLP40: Thylakoid Lumen Proteins of 18.3 and 40 kDa
TPR: tetratricopeptide repeat
TRX: thioredoxin
VAR1 and VAR2: Yellow Variegated 1 and 2, also known as FtsH5 and FtsH2, respectively
VIPP1: Vesicle-Inducing Protein in Plastids 1
YCF48: hypothetical chloroplast reading frame number 48.
